# Quantifying the dynamics of peak disruption in scientific careers

**DOI:** 10.1038/s41598-025-95264-8

**Published:** 2025-03-28

**Authors:** Mingtang Li, Giacomo Livan, Simone Righi

**Affiliations:** 1 Department of Computer Science, University College London, 66-72 Gower Street, London, WC1A 6EA UK; 2 Department of Physics, University of Pavia, Via Bassi 6, 27100 Pavia, Italy; 3 Department of Economics “Marco Biagi”, University of Modena and Reggio Emilia, Via Berengario 51, 41100 Modena, Italy; 4 Sezione di Pavia, Istituto Nazionale di Fisica Nucleare, Via Bassi 6, 27100 Pavia, Italy

**Keywords:** Scientific disruption, Scientific careers, Academic impact, Disruptive innovation, Engineering, Physics

## Abstract

We examine the disruption of researchers with long-lived careers in Computer Science and Physics. Despite the epistemological differences between such disciplines, we consistently find that a researcher’s most disruptive publication does not occur at random during their career, as it cannot be explained by a null model. Such publication is accompanied by a peak year in which researchers publish other work that exhibits a higher level of disruption than average. Through a series of linear models, we show that the disruption achieved by a researcher during their peak year is higher when it is preceded by a long period of focus and low productivity. These findings are in stark contrast with the dynamics of academic impact. In these dynamics, researchers are incentivized by the prevalent paradigms of scientific evaluation to pursue high productivity and incremental—less disruptive—work, as evidenced by extensive literature.

## Introduction

Newcomers to academia often harbor the aspiration to make breakthroughs in their research field. Although a researcher may leave a lasting mark in their area with a single publication, in today’s academic environment building a long-lived career requires a demonstrable ability to produce a steady stream of works, consistently published in reputable peer-reviewed venues. In this respect, a quantitative understanding of disruptive innovation ultimately boils down to identifying patterns that may reveal how a researcher’s disruptiveness evolves throughout their career, as characterized by the sequence of their publications. Is the ability to disrupt roughly constant or does it instead peak at a certain career stage? And, if so, which factors are conducive to periods of high disruptive innovation in a scientific career?

Answering these questions is especially important in today’s highly competitive research ecosystem, marked by ever-increasing volumes of publications competing for attention^1^. In fact, such an environment incentivizes researchers to maximize the impact of their scientific work. On the one hand academic impact is a multifaceted concept, encompassing various dimensions including – among others – the plausibility^2,3^, originality^4^, scientific value^5–7^, and societal value^8–10^ of scientific publications. On the other hand, however, current research evaluation practices mostly operationalize academic impact in terms of bibliometric impact, i.e., the amount of citations that published scientific work receives from other publications^11–13^. This, in turn, has led to the proliferation of citation-based bibliometric indicators that seek to quantify different aspects of a paper’s or a researcher’s ability to attract citations^14–16^.

There is an abundance of literature showing that equating academic impact with bibliometric impact shapes the career choices of researchers in at least two ways that are very consequential to disruptive innovation. First, it incentivizes the pursuit of conservative research in order to publish ‘safer’ contributions^17,18^. For instance, despite some controversy^19^, a large body of literature demonstrates that cross-disciplinary research comparatively attracts fewer citations than incremental research that seeks to develop an already well-established field^20,21^. As much as such an approach may be beneficial to the career progression of individual researchers, on a collective level it dramatically stifles risk-taking^22^, which is crucial for scientific breakthroughs. Second, it incentivizes excessively high productivity (i.e., the so called ‘publish or perish’ culture^23^), resulting in a loss of sustainability in scientific work^24^.

One key reason bibliometric indicators of impact are so widely employed is that they are easy to use and calculate. The same cannot be said for metrics quantifying disruptive innovation. While the study of disruptive innovation has a long history in, e.g., Economics^25,26^, Management^27^, and Anthropology^28^, the development of indicators for disruption is a relatively recent endeavour. Early efforts to quantify scientific breakthroughs introduced the renowned novelty metrics, which evaluate scientific publications by determining how typical/atypical the references of a paper are^29^. A similar method proposed by Wang *et al.* measures novelty by identifying previously unseen journal pairs in a paper’s bibliography^30,31^. Another approach examines the degree of citation similarity between a focal paper and its preceding works^32,33^. However, these metrics have been shown to underestimate research in well-defined fields or prove inadequate for interdisciplinary analysis^33,34^, making them less suitable for our study.

An alternative measure – known as the CD index, or disruption score^35,36^ – overcomes these issues, and has demonstrated its ability to distinguish between disruptive and developmental contributions. Its robustness has been validated across data from scientific publications, patents, and software products^35–37^. Recent studies have explored the relationship between disruption scores and citation impact^38,39^, indicating that they essentially capture different types of information^39^. The distinction between the two metrics enables a comparative analysis in our study. Moreover, a proper specification of the disruption score is crucial for our research^40^. Although the scientific community has not reached a consensus on the most effective formulation of the disruption score, it recommends a set of best practices for using this metric^41^. In line with these guidelines, we will provide a detailed explanation of our approach to the disruption score in this analysis.

Leveraging on the disruption score, this paper aims to quantify the tension between disruptive innovation and citation impact. Specifically, we hypothesize that high levels of disruption require to devote effort to specific projects or research questions over extended periods of time, i.e., ultimately, low productivity. We will validate such hypothesis by tracking the publication sequence of a large pool of researchers in Computer Science and Physics with long-lived careers. First, we will demonstrate the existence of specific career stretches in which researchers are consistently more disruptive than in the rest of their career. Second, we will relate the productivity of a researcher in the run-up to such periods with the level of disruption achieved during them. Our results will show that researchers experience a ‘magical year’ characterized by disruptive publications, and that such publications are more disruptive when published after a period of low productivity.

Given our research purpose, the career dynamics of researchers are also highly relevant to our analysis. In this context, previous studies highlight the importance of age for scientific creativity. While major scientific breakthroughs were often achieved by young researchers in the early days of science, modern studies indicate that the average academic age to achieve these breakthroughs has gradually been delayed^42,43^. This finding is supported by^44^, which states that researchers typically achieve an optimal balance between creativity and practical experience in the middle stage of their careers. ^44^ also emphasizes that significant career milestones, such as the first major work and peak contributions, are key to understanding the careers of researchers. In addition to academic age, factors such as discipline, research field maturity, and academic position also affect scientific creativity^42,45,46^. It is also worth mentioning that these studies primarily use citations and productivity as measures of scientific achievements.

## Results

We utilize publication and citation data in Computer Science and Physics obtained from the AMiner citation network dataset (version 12) and the Web of Science database, respectively (see Methods). We select researchers in these two disciplines according to the filtering criteria of^47^, establishing a pool of 27,641 and 34,526 researchers with long-lived careers in Computer Science and Physics, respectively (see Methods).

To quantify the disruption dynamics of scientific careers, we calculate the disruption score for each paper authored by our selected researchers (see Methods). Given the fact that a scientific career is fundamentally characterized by the sequence of papers published by the researcher, the disruption dynamics of a researcher can be represented as the sequence of disruption scores associated to their publications. Moreover, a key feature of the disruption score is its ability to broadly rank papers based on their disruption levels. This feature enables us to analyze scientific careers by assessing the years in which a researcher’s publications are the most disruptive in their career and examining their relationship with other years of activity and published works.

In accordance with the best practices for using the disruption score^41^, we now present our methodological decisions regarding the application of this metric and explain the reasons. Most importantly, we compute the disruption score of a paper using its full record of subsequent papers, rather than just those published in the following 5 years, as often done in the literature^36,37^. We do this for three reasons. First, using a longer citation window circumvents the issue of delayed recognition for disruptive research^30^. Moreover, the disruption score of a paper does not increase monotonically with time, but varies with the citation behaviours of its subsequent papers. Incorporating the full publication history that follows it can provide a more accurate reflection of a paper’s true disruption level. Third, a paper’s disruption score may be biased upwards when it is computed based on a small number of citations. To mitigate this issue, we use the complete publication record to include more subsequent papers in the calculation of the disruption score.

Nevertheless, our decisions may subject the disruption score to other biases. To address the issue of citation inflation^48^, we standardize the disruption score of each paper with respect to its year of publication (see Methods), and corroborate all our results with the standardized disruption score. Moreover, the value of the disruption score is sensitive to the number of backward and forward citations^49^. To control for this bias, we validate all of our results using the CD$\vphantom{0}_5$ metric (i.e., the disruption score computed only from papers published in the 5 years following the focal paper) as an additional robustness check throughout our analysis.

As a further validation of our results against potential biases from books, reviews, and self-citations, we select the most disruptive and well-cited papers in our datasets by setting a threshold of $n_i > 100$ (see Eq. (2) in Methods). Applying this threshold, we identify 19,938 papers in Computer Science and 11,867 papers in Physics, corresponding to 7,547 and 8,576 researchers, respectively. We also repeat our analysis with this pool of the most disruptive researchers in both disciplines.

### The non-randomness of peak disruption

We begin our study by measuring how disruption – as quantified by the disruption score – evolves throughout a researcher’s career. We first identify the year in which a researcher publishes their most disruptive paper, i.e., their publication with the highest disruption score. In the following, we shall refer to such a year as the ‘peak year’. Following the analysis by Sinatra *et al.* on the randomness of scientific impact during careers^50^, we then partially randomize a researcher’s disruption trajectory across their publication history by keeping the publication dates of their papers intact while randomly reassigning the disruption scores associated to such publications.

**Fig. 1:**
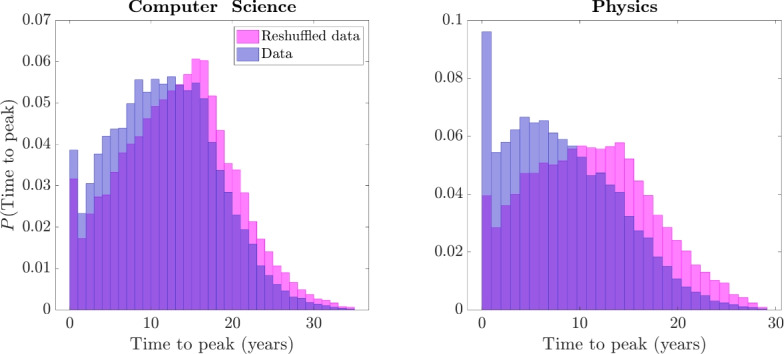
Normalized histograms of the time (measured in years) to reach their peak in disruption for researchers in Computer Science (left) and Physics (right) obtained from the original (purple) and randomized (pink) data.

We calculate the number of years between a researcher’s first published paper and their peak year both in the original and in the randomized data. The results are presented as histograms in Fig. 1. In both Computer Science and Physics, the distributions derived from the original and randomized data are significantly different ($p < 0.01$ for both disciplines, two-sided Kolmogorov-Smirnov – KS-test). Researchers in both disciplines reach their disruption peak year earlier than expected based on the null model ($p < 0.01$ in both disciplines, Mann-Whitney U test – MWU test). It should be noted that Fig. 1 is obtained by comparing the real data with a single instance of the null model. However, the differences observed across multiple independent instances of the null model are also negligible (see Supplementary Figure 1). Overall, our results indicate that the disruption peak year in a scientific career does not occur at random.

To validate our results, we first examine the position of the most disruptive paper in the publication sequence of researchers, i.e., identifying the number of papers (rather than the time taken) to reach the disruption peak year. This analysis yields equivalent results in both disciplines ($p < 0.01$ in both disciplines, KS test), as shown in Supplementary Figure 2. We also replicate our results using the CD$\vphantom{0}_5$ metric and the pool of most disruptive researchers, still finding consistent results ($p < 0.01$ in both disciplines, two-sided KS-test), see Supplementary Figures 3-4. To control for the temporal bias of the disruption index, we further employ the standardized disruption score to validate our results. As depicted in Supplementary Figure 5, while we continue to observe the non-randomness of peak disruptions, the precise timing of these peaks changes. Specifically, researchers in Computer Science now achieve their disruption peak year later than the randomized null model ($p < 0.01$, MWU test). In Physics, there is an excess of researchers who reach their peak disruption within the first two years of their career, as well as those who peak later than the randomized baseline. This pattern results in an inconclusive MWU test for physicists in our analysis ($p = 0.238$, MWU test).

We ought to acknowledge the inconsistency in the precise timing of the disruption peak year between our original and standardized results. We attribute this inconsistency to the temporal effect of the disruption score. Specifically, in our original results, researchers tend to publish their most disruptive papers early on because their careers follow a general trend of decreasing disruption over time^37^. This attribution is grounded on the only difference between our original and standardized results: whether or not the temporal effect of the disruption score has been accounted for. Despite the inconsistency, it is important to note that regardless of whether we control for the temporal effect, the corresponding results still deviate from the randomized null model. Therefore, we conclude that peak disruption – unlike peak impact^50^ – does not occur entirely at random in a researcher’s career.

### Characterizing disruption peak year

Given the non-randomness of peak disruption, we now proceed to investigate whether a researcher’s peak year presents peculiar statistical regularities. To this end, we address two research questions in this analysis: (1) Does the peak year exhibit a higher level of disruption compared to other phases of a career? If so, (2) Does the peak year happen at the expense of other published papers being less disruptive? We hypothesize that the peak year is generally characterized by a higher disruption level, rather than by just a single disruptive paper published among less disruptive ones.

To test such conjecture, we split the careers of researchers into ‘before peak year’, ‘peak year’, and ‘after peak year’ phases. After that, we calculate the average disruption level achieved by our pool of researchers in all phases. Specifically, we compute the average ‘peak year’ (PY) disruption level for a researcher using all their papers published during the peak year. The disruption level of other career phases are calculated for 5, 4, 3, and 2 years before and after the peak year (collectively referred to as B/APNY, N $= 5, 4, 3, 2$, see Supplementary Table 1 for more information), with the aim of mitigating biases arising from the differences in lengths of the before/after peak phases.

**Fig. 2:**
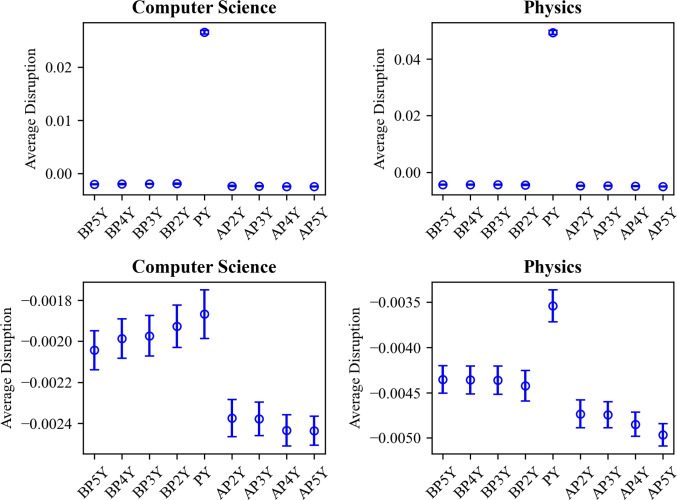
Average disruption levels achieved by researchers in Computer Science (left column) and Physics (right column) across the ‘before peak year’ (BP5Y-BP2Y), ‘peak year’ (PY), and ‘after peak year’ (AP2Y-AP5Y) phases. The PY phase is computed both with the peak disruption paper included (top row) and excluded (bottom row) from the analysis. Error bars represent standard errors.

We then compare the disruption level of the three phases and present the result in Fig. 2. In this figure, we choose to show standard errors rather than standard deviations because the latter are rather large and would significantly diminish the visual interpretability of the plots. We find that the distributions of disruption scores in the BPNY and APNY phases are significantly different from those in the peak year ($p < 0.01$ in all cases, two-sided KS tests). More importantly, the average disruption score is much higher in the peak year than in the other two phases, as supported by both the MWU-test ($p < 0.01$ in all cases for both disciplines) and Cohen’s *d* analysis ($\sim 0.65$ in Computer Science and $\sim 0.53$ in Physics, see Supplementary Table 2). Our findings suggest that the peak year represents a special career stage for researchers, as its disruption level is significantly higher than that of other phases in a scientific career.

Building upon question (1), we now investigate the second research question in our analysis. Specifically, we re-examine our comparison across the three phases, with the disruption level for the ‘peak year’ (PY) phase computed excluding the paper responsible for the peak itself. As illustrated in Fig. 2, we still observe that the disruption level of the peak year is higher than that of the other two phases (KS-test, $p < 0.01$ in all cases; MWU-test, $p < 0.01$ in both disciplines, except $p = 0.043$ between BP2Y and PY in Computer Science). Although the effect size is not large according to the Cohen’s *d* analysis, our results collectively indicate that the publication of the most disruptive paper does not come at the expense of other papers published during the peak year being less disruptive in the careers of researchers.

Notably, in constructing the ‘peak year’ phase for question (2), we focus on researchers with two or more publications during their peak year, i.e., with at least one more paper published in addition to their most disruptive work. This criterion reduces the number of targeted researchers in both disciplines ($N_\textrm{CS}$ from 27,641 to 18,640, $N_\textrm{PHY}$ from 34,526 to 26,164). To better illustrate the changes in our dataset, we present the distribution of the number of papers published during the peak year in Supplementary Figure 6. For comparison, we also include the distribution of the number of papers published five years before and after the peak year in the same figure. Our analysis reveals that the number of publications in the peak year follows a power law distribution, consistent with the distribution observed in the comparison baseline.

As for robustness checks, we first repeat the above analysis with both the CD$\vphantom{0}_5$ metric and the standardized disruption score, finding equivalent results, see Supplementary Figures 7-8. Then, we replicate these results within the pool of most disruptive researchers in both disciplines. We observe that while our results remain consistent in Physics, in Computer Science the peak year disruption level (excluding the peak paper) is not the highest in comparison to other phases. Nevertheless, the peak year still remains the most disruptive one for researchers in Computer Science when peak papers are included in the analysis, see Supplementary Figure 9.

Furthermore, we address the potential issue of the average disruption score not being a representative indicator of a researcher’s disruption throughout their career. In order to do that, we employ an alternative method for representing publications in the before and after the peak year phases. Precisely, we gather papers contributing to B/APNY in our dataset and conduct a direct comparison of the score distributions of these papers. Our results remain consistent in both disciplines, as shown in Supplementary Figure 10.

### Determinants of peak-year disruption

Having established the existence of a ‘magical year’ characterized by generally higher levels of disruption, and having established that it does not occur at random in a researcher’s career, we now seek to establish its relationship with the effort devoted to achieve that peak. In order to do so, we quantify the overall disruption of a researcher at their peak year as the average disruption score of their papers published during such year, including the paper responsible for the peak in disruption.

We calibrate a series of linear models to investigate the relationship between the level of disruption achieved by a researcher in their peak year and a set of variables aimed at quantifying the effort put into the scientific work published during that year. We aim to capture the trade-off between the incentive to publish frequently – i.e., productivity – and the ability to produce disruptive contributions. Our hypothesis is that spending more time working on a paper should increase its likelihood of being disruptive. We quantify effort (see Methods) as the average time (measured in years) spent on a paper published during a period of interest. More specifically, we calculate the ratio between a variable we shall refer to as ‘time devoted’ and the number of papers published by a researcher (i.e., their productivity) during the period of interest. Time devoted is defined as the number of years between a researcher’s last publication before the period of interest and the final year of that period.

We also define corresponding relative quantities, i.e., measures of effort, time devoted, and productivity computed as the ratio between such quantities during a period of interest and over the entire career of a researcher. In Supplementary Figure 11, we plot the distributions of such quantities when using the peak year as the period of interest. In the following, we only present results obtained based on such relative quantities, given that the results obtained from the corresponding absolute quantities are qualitatively equivalent (see Supplementary Tables 7-10).

In three distinct models, we measure the linear relationship between a researcher’s disruption during their peak year and the relative effort, relative productivity, and relative time devoted associated with that year. In each model we control for the same additional factors. We include the time to reach the peak year (measured in years) and the calendar year corresponding to the peak year in order to control for possible temporal effects. We also include the researcher’s number of coauthors in their papers published during the peak year to control for team size and network effects. Lastly, we include the average disruption score of papers published in the two years before the peak year to control for idiosyncrasies in the ability to disrupt. The regression model is explicitly defined as follows:

1 $$\begin{aligned} \begin{aligned} \text {Peak}\ \text {year}\ \text {disruption}\ \text {level}&\ = \beta _0 \\&\ +\ \beta _1 * \text {Key}\ \text {variable} \\&\ +\ \beta _2 * \text {Avg. num. of coauthors} \\&\ +\ \beta _3 * \text {Avg. prev. disruption} \\&\ +\ \beta _4 * \text {Peak year} \\&\ +\ \beta _5 * \text {Time to peak} \end{aligned} \end{aligned}$$

where ‘Key variable’ represents relative effort, relative productivity, relative time devoted and their corresponding absolute quantities, and $\beta _0$, $\beta _1$, $\beta _2$, $\beta _3$, $\beta _4$, $\beta _5$ are the regression coefficients.

**Fig. 3:**
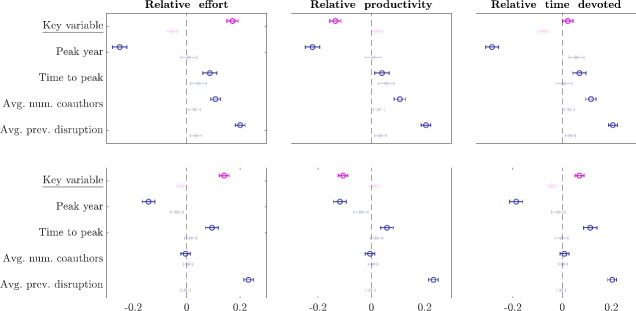
Coefficients of linear regression models for peak year disruption (circles, bright colours) and impact (crosses, dim colours) in Computer Science (top row) and Physics (bottom row). Each column displays results obtained with a different key variable of our analysis, i.e., relative effort (left), relative productivity (center), relative time devoted (right). Magenta (blue) symbols refer to the coefficients associated with key regressors (control variables) in each model. Key regressors are highlighted in the plot’s titles. Error bars represent standard errors multiplied by three. From left to right, in Computer Science models for disruption have an $R^2$ of 0.142, 0.132 and 0.116, while models for impact have an $R^2$ of 0.009, 0.007 and 0.011. In Physics, models for disruption have an $R^2$ of 0.086, 0.078 and 0.071, while models for impact have an $R^2$ of 0.001, 0.001 and 0.002.

The results of the aforementioned models are illustrated in Fig. 3. Both in Computer Science and Physics relative effort contributes positively to the disruption level achieved during the peak year, and the same holds for relative time devoted. Contrastingly, relative productivity is found to be detrimental to peak year disruption. Overall, we interpret these results as a clear sign that peak disruption improves when preceded by a period of focus on the work that will be eventually published during the peak year, and working on less papers around the peak years yields higher peak disruption.

A priori, one could not rule out that the disruption score provides similar information to the citation impact of a paper. In order to test this hypothesis, we calibrate the linear models described above with the same set of independent variables and impact as dependent variable. We measure the latter with the accumulated citations of peak year papers over the first 5 years after their publication. We find the explanatory power of such models to be quite low. Yet, they still provide important insight on the correlations between our effort-related variables and scientific impact. In fact, we find that higher relative productivity contributes to a higher impact, whereas relative effort and relative time devoted have the opposite effect (see Fig. 3). Similar considerations apply when considering the corresponding absolute quantities. As an example, Table 1 reports the regression results for peak year disruption in Computer Science. Regressions of other models can be seen in Supplementary Tables 7-10.

The control variables in our model also contribute to explain the determinants of peak disruption. Indeed, we find that researchers who reached their peak in more recent years achieved – on average – lower disruption levels, in line with various studies that have evidenced how science has become less disruptive over time^37,51–54^. After controlling for such an effect, we observe a positive relationship between peak disruption and the time to reach the peak. Our results also show that – in both disciplines – the more disruptive a researcher’s previous papers are, the more disruptive they will be in the peak year. Additionally, publishing with more co-authors has a positive influence on the peak year disruption level in Computer Science, but not in Physics.

As a concrete example of the meaning of our regression results, consider the following pair of two researchers in Computer Science, which we will refer to as A and B. The career trajectories of A and B are very similar: both began their careers at the same time and took 8 years to reach their disruption peak in 1990. They demonstrated a similar level of pre-peak disruption, and collaborated with the same number of coauthors in their peak year. The main difference is that A’s relative effort and relative time devotion in the peak year are 5.8 and 2.1 times greater than those of B, but with only one-third of B’s relative productivity. Correspondingly, A’s peak year disruption level is 3.3 times higher than that of B, yet A received only 13 citations within 5 years, compared to B’s 44 accumulated citations in the same period. If we evaluate researchers A and B solely by citation counts, B is undoubtedly superior to A. However, when evaluating their performance through both citation counts and disruption scores, each researcher exhibits unique strengths: while B is more productive and achieves a higher citation impact, A devotes more time and effort in each publication, resulting in more disruptive scientific outcomes.

For robustness checks, we calibrate regression models with the above variables computed in the two years before the peak as additional controls. In addition, we construct linear models using CD$\vphantom{0}_5$, the standardized disruption scores, and the pool of most disruptive researchers. The results we obtain in these models are qualitatively equivalent to the main results presented here (see Supplementary Tables 11-26).

To control for paper length and the influence of review papers on our analysis, we incorporate the logarithm of the average number of references as an additional independent variable, as illustrated in Supplementary Tables 27-30. In this analysis, the relative productivity in Computer Science becomes statistically insignificant in the impact regression, yet all other results, including productivity regressions, still remain consistent. Moreover, we observe that a higher number of references, i.e., longer paper lengths, is positively associated with impact in the peak year but negatively affects peak year disruption levels.

**Table 1:** Computer Science: Regressions for peak year disruption level with peak year data.

Indep. Variables	Model 1	Model 2	Model 3	Model 4	Model 5	Model 6
Relative effort	0.172*** (0.007)					
Effort		0.137*** (0.007)				
Relative productivity			$-$0.135*** (0.007)			
Productivity				$-$0.121*** (0.007)		
Relative time devoted					0.023*** (0.007)	
Time devoted						0.021*** (0.007)
Avg. num. of coauthors	0.108***	0.099***	0.107***	0.103***	0.117***	0.117***
	(0.006)	(0.007)	(0.006)	(0.007)	(0.007)	(0.007)
Avg. prev. disruption	0.201***	0.209***	0.205***	0.209***	0.206***	0.206***
	(0.006)	(0.006)	(0.006)	(0.006)	(0.007)	(0.007)
Peak year	$-$0.251***	$-$0.256***	$-$0.221***	$-$0.256***	$-$0.285***	$-$0.272***
	(0.009)	(0.009)	(0.009)	(0.009)	(0.010)	(0.009)
Time to peak	0.087***	0.067***	0.040***	0.070***	0.070***	0.057***
	(0.009)	(0.009)	(0.009)	(0.009)	(0.010)	(0.009)
N	20,980	20,980	20,980	20,980	20,980	20,980
$R^2$	0.142	0.133	0.132	0.129	0.116	0.116

*p < 0.1; **p < 0.05; ***p < 0.01.

## Discussion

We examine the career dynamics of researchers from the perspective of their disruptive innovation, measured in terms of the disruption score^35,36^. We find that peak disruption does not occur at random in a researcher’s career, and that the time it takes for most researchers to reach their peaks varies depending on their discipline. This is in contrast with the so called ‘random impact rule’^50,55^, which states that each paper in a researcher’s career is equally likely to become their most cited publication.

Research on career analysis indicates that individuals tend to experience ‘hot streak’ periods in which they enjoy sustained success in their work^55–57^. Our results demonstrate that a similar effect holds for disruptive innovation. In fact, we show that the disruption peak year represents a special phase of high disruption in researchers’ careers. Such a phase is characterized not just by one very disruptive paper, but by a series of papers that are more disruptive than average. In other words, our findings show that papers published during the peak year enjoy a synergistic effect, i.e., the paper responsible for the peak does not drain a researcher’s resources away from other publications.

Regarding the precise timing of peak disruption, we show that disruption peaks in scientific careers happen - on average - earlier than one would expect based on a ‘random disruption rule’, i.e., a null model that randomizes disruption across the sequence of publications in a researcher’s career. However, this result should not be simply interpreted as younger researchers being more disruptive than senior ones. In fact, we observe a significant proportion of researchers who reach their peak years later than expected after controlling for the temporal effect of the disruption index. At the same time, our regression analysis indicates that disruption peaks that happen later in a career tend to be higher. These results together suggest that the intuition that senior scientists tend to be less disruptive is likely to be influenced by the overall decrease in scientific disruption, which we consistently detect in our models. In further contrast to this intuition, we find that accumulating research experience can be important for disruptive innovation, as evidenced by the fact that researchers achieve their peaks later in their career more often than expected, and late peaks are accompanied by higher levels of disruption (after controlling for the covariates included in our analysis).

Having determined that peak disruption does not happen at random, and it is part of a special period of high disruption, we then shine light on the determinants of the magnitude of such a peak. To this end, we run a series of regression models where the magnitude of the peak disruption is regressed against various measures that proxy the effort infused by an individual in their peak year papers, and various measures of productivity. We find that devoting more time and effort to the research published in the peak year has a positive correlation with the disruption level achieved during it, while measures of productivity have a negative correlation with it. One possible explanation for this result is that working on many papers simultaneously may drain researchers of their energy and ideas. Another potential reason lies in the so called ‘salami slicing’ strategy in scientific research^58^, which involves splitting findings from the same studies into multiple publications to maximize impact in terms of expected citation volume.

Such a tension between disruption and impact is well captured by our models, which show that those two dimensions have opposite correlations with the aforementioned proxies of effort and productivity. Disruption thrives when more effort is infused into scientific work, i.e., experiencing periods of focus and low productivity. Conversely, low productivity is detrimental to impact. While one should not read too much into the latter result due to the reduced amount of variation explained by our models for citation impact, these findings strongly suggest that disruption and citations follow very different patterns, supporting the claims by Aksnes et al.^4^ and Li et al.^39^ that such two concepts should indeed be distinct.

Our work is subject to certain research limitations. In this paper, we primarily use the disruption score as a proxy for disruptive innovation. This methodological choice — despite our efforts to check its robustness — may still expose our analysis to the inherent limitations of the disruption score, including biases from citation inflation^48^, the number of backward and forward citations^49^, and the specification of the disruption score^40^. Additionally, due to constraints of our dataset, we cannot distinguish between peak years when researchers explore a variety of topics and those when they study predominantly in closely related fields. We are also unable to further refine our analysis to the sub-fields of each discipline. Moreover, contextual factors such as collaboration networks can influence the performance of researchers^47^. We suggest that future studies should address the connection between these factors to further reveal the underlying mechanism of disruptive innovation.

Researchers are frequently evaluated on bibliometric indicators of citation impact, e.g., for tenure decisions or promotions^59^. Our analysis points to the fact that they should also be evaluated for their ability to disrupt, as the latter might provide a very different perspective on their work. Moreover, our findings indicate that a long-lived academic career is important for researchers to achieve higher disruptions. Scientific policymaking should therefore promote career stability in order to allow more researchers to remain in academia.

## Methods

### Data

We collect publication and citation data for Computer Science and Physics from the AMiner citation network dataset (version 12) and the Web of Science database, respectively. The AMiner dataset contains papers from the 1960s to 2020 from DBLP, ACM, MAG, and other major sources in Computer Science^60^, and it contains a total of 4,894,081 papers and 45,564,149 citations between them. The AMiner dataset has been employed in a number of science of science studies, including some published in top-tier venues^61–63^. For Physics papers, we extract data from the Web of Science (WOS) database. We extract the publications of a specific selection of researchers (see below) and the citation network of their publications. Overall, we gather a total of 1,619,039 papers and 12,621,175 citations between them from 1985 to 2020.

While the Computer Science AMiner dataset performs strict name disambiguation method by analyzing citation relationships and coauthorship networks, the Physics WOS database does not maintain unique author identifiers. Therefore, in order to associate authors to their publications, we apply a state-of-the-art approach proposed by Caron and van Eck to disambiguate author names^64^. Specifically, this method computes a similarity score between pairs of authors based on a series of attributes, including ORCID identifiers, names, affiliations, emails, coauthors, grant numbers, subject categories, journals, self-citations, bibliographic coupling, and co-citations. The higher the similarity score between two authors, the more likely they are to be considered the same individual. A recent study has demonstrated that this method is able to outperform other unsupervised disambiguation methods^65^, with precision and recall scores higher than 90%.

In our study, we calculate disruption scores only for papers published before 2016, thereby allowing papers in our dataset to accumulate citations for at least 5 years. We also exclude papers with fewer than 5 references in their bibliography, as these papers are likely to suffer from missing value problems^66^ and their disruption scores would become outliers in our analysis. To create our researcher dataset, we set filtering criteria in line with^47^, selecting only researchers with long-lived careers. The selected researchers are those who began their careers between 1980 and 2000, had at least 20 years of career span, published at least 10 papers, and published at least one paper in every five-year period. These filtering criteria give us a total of 27,641 and 34,526 researchers in Computer Science and Physics, respectively.

### The disruption score

In order to address our research questions, we adopt the disruption score to quantify the disruption level of each paper in our datasets. Such a metric can effectively distinguish between disruptive and developmental publications, and its robustness has been validated against data from scientific papers, patents, and software products^35,36^. The key idea of this indicator is that a highly disruptive paper will eclipse attention towards preceding work in the same fields, i.e., subsequent publications will tend to cite such a paper more than the references in its bibliography.

More specifically, consider a citation network built around a focal paper, its references (previous papers) and subsequent papers. The subsequent papers can be classified into those that cite only the focal paper, those that cite both the focal paper and previous papers, and those that cite only the previous papers. Let us assume that the number of papers in each group is $n_i$, $n_j$ and $n_k$, respectively. Then the disruption score is given by

2 $$\begin{aligned} D = \frac{n_i - n_j}{n_i + n_j + n_k} \end{aligned}$$

where $n_i - n_j$ measures the extent to which the focal paper has eclipsed attention towards previous papers, and $n_i + n_j + n_k$ represents the total number of subsequent papers in the entire citation network.

Based on the above definition, the disruption score ranges from -1 to 1. A positive score suggests that the focal paper attracts more attention from subsequent papers than its references, which means that the focal paper is more disruptive. If a focal paper is disruptive enough, then its disruption *D* should be close to 1. Similarly, a negative score means that the focal paper is likely to be a developmental piece of work. The closer the score to -1, the more developmental the paper will be. Therefore, the disruption score enables us not only to quantify each paper’s disruption level but also to compare the disruption level among different papers.

As mentioned in the Results section, here we explain in detail the procedure of standardizing the disruption score. We standardize the disruption score of each paper with respect to its year of publication, i.e., we group papers in our dataset based on their year of publication and standardize using the mean and standard deviation of that year’s distribution of disruption scores (i.e., transforming into z-scores). In this paper, we corroborate all of our results using the standardized disruption scores.

### The effort measure

In the ‘Determinants of peak-year disruption’ section, we introduce a new metric called ‘effort’ to quantify the time a researcher dedicates to each paper published over a specified period. Essentially, the effort measure indicates the average time allocated to each publication within that period. The formula for the effort measure is simply given by

3 $$\begin{aligned} \mathrm {Effort\ (over\ a\ period)}\ =\ \frac{\mathrm {time\ devoted}}{\textrm{productivity}} \end{aligned}$$

where ‘time devoted’ refers to the time interval between the publication of the last paper before the specified period and the end of that period. ‘Productivity’ represents the number of publications within the specified period.

As a practical example of the effort measure, consider a researcher who publishes two papers in 2005, three papers in 2007, and five papers in 2008. The researcher’s effort during the 2007-08 period is calculated as follows. First, the ‘time devoted’ for the specified period (2007-08) is three years, as the last paper before the period is published in 2005 and the specified period ends in 2008. Second, the productivity within this period is eight, as given by the total number of publications from 2007 to 2008. Hence, the effort over the 2007-08 period is 3/8. To interpret the effort metric, the researcher publishes eight papers over three years, so each paper takes 3/8 years to prepare.

## Supplementary Information

Supplementary Information.

## Data Availability

All codes used in this study are available from the corresponding author upon request.

## Data availability

The AMiner dataset used in the paper to collect data about publications and citations in Computer Science is publicly available at https://www.aminer.cn. The publication and citation data used for Physics were obtained from Web of Science.
