# Supplementary material for: Quantifying the dynamics of peak disruption in scientific careers
Source: Sci Rep. 2025 Mar 28;15:10812. doi: 10.1038/s41598-025-95264-8 (PMC11953407; doi:10.1038/s41598-025-95264-8)
Supplement: Supplementary file 1 — Supplementary Information. [file 41598_2025_95264_MOESM1_ESM.pdf]

Supplementary Information: Quantifying the dynamics of peak disruption in scientific careers

Mingtang Li[1*], Giacomo Livan[1,2†] and Simone Righi[1,3†]

[1]*Department of Computer Science, University College London, 66-72 Gower Street, London, WC1E 6EA, United Kingdom*

[2]*Systemic Risk Centre, London School of Economics and Political Sciences, Houghton Street, London, WC2A 2AE, United Kingdom*

[3]*Department of Economics, University Ca'Foscari of Venice, Fondamenta S. Giobbe 873, Venezia, 30121, Italy*

[*]*Corresponding author E-mail: g.livan@ucl.ac.uk*

The PDF file includes:

- Supplementary Notes 1 to 4
- Supplementary Figures 1 to 11
- Supplementary Tables 1 to 31

Supplementary Note 1 The non-randomness of scientific disruption: additional details

We demonstrate the non-randomness of scientific disruption by comparing the distribution of the time taken by researchers to reach their disruption peaks based on their original and reshuffled sequences of publications. Here we further validate this result in the following ways.

First, we compare our original data with 100 instances of the null model to quantify the size of the fluctuations in the randomized data. The results are presented in Supplementary Figure 1. We then compare the distributions of the numbers of papers published by researchers before their peak year. The comparison of distributions is illustrated in Supplementary Figure 2. Third, we replicate our results using the CD_5 metric, as shown in Supplementary Figure 3. Fourth, we repeat the experiment in the main text with our pool of most disruptive researchers. The results are depicted in Supplementary Figure 4. Lastly, we conduct the comparison using the standardized disruption score, as illustrated in Supplementary Figure 5. In both disciplines, the distributions obtained from the original and randomized data are significantly different ($p < 0.01$ in all cases, two-sided KS test), and our findings are consistent across all the validation methods.

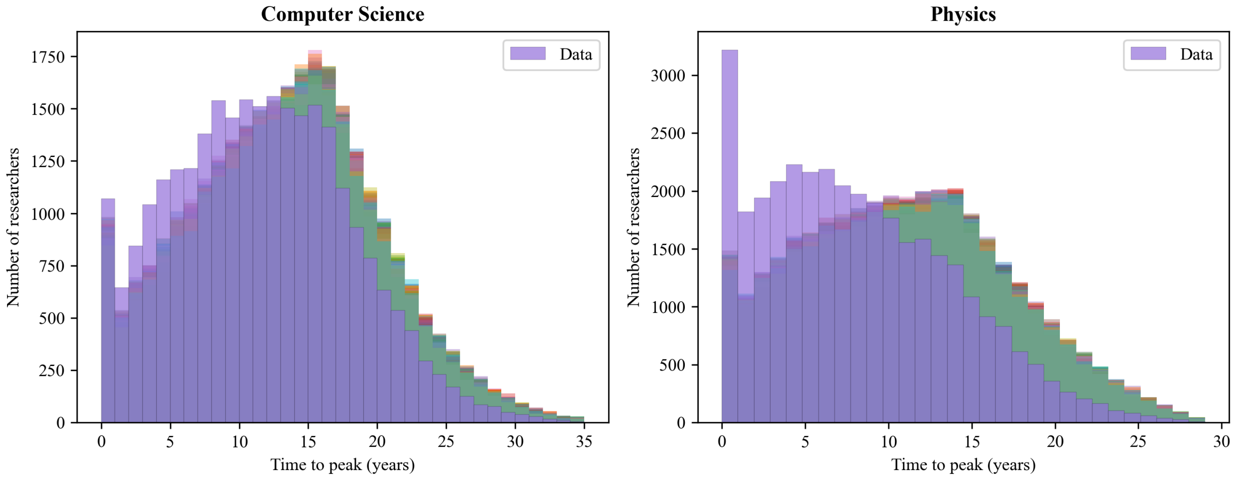

Supplementary Figure 1. Histograms of the number of years spent by researchers to reach their peak year in Computer Science (left) and Physics (right), obtained from the original data (purple in front) and 100 randomized null models (other colors in back).

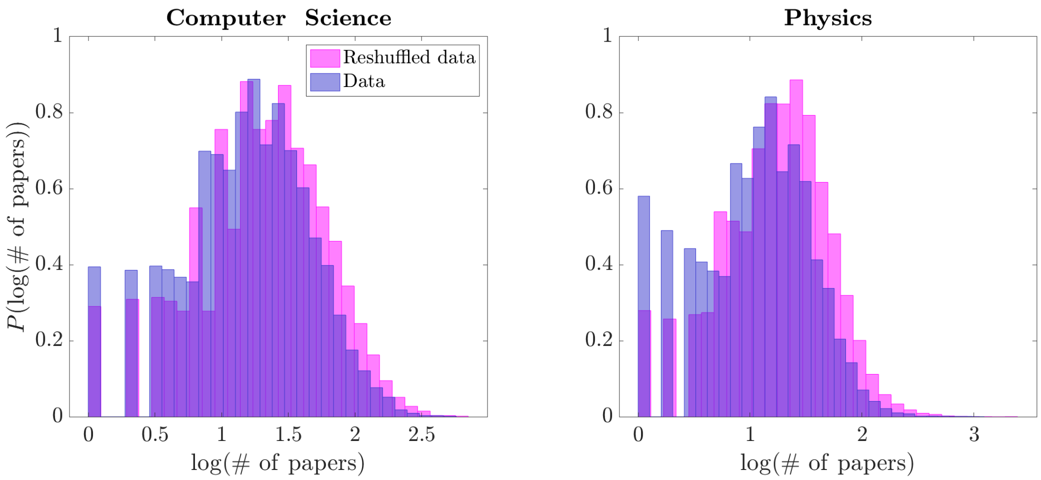

Supplementary Figure 2. Normalized histograms of the number of papers published by researchers before their peak year in Computer Science (left) and Physics (right) based on the original (purple) and randomized (pink) data after log transformations.

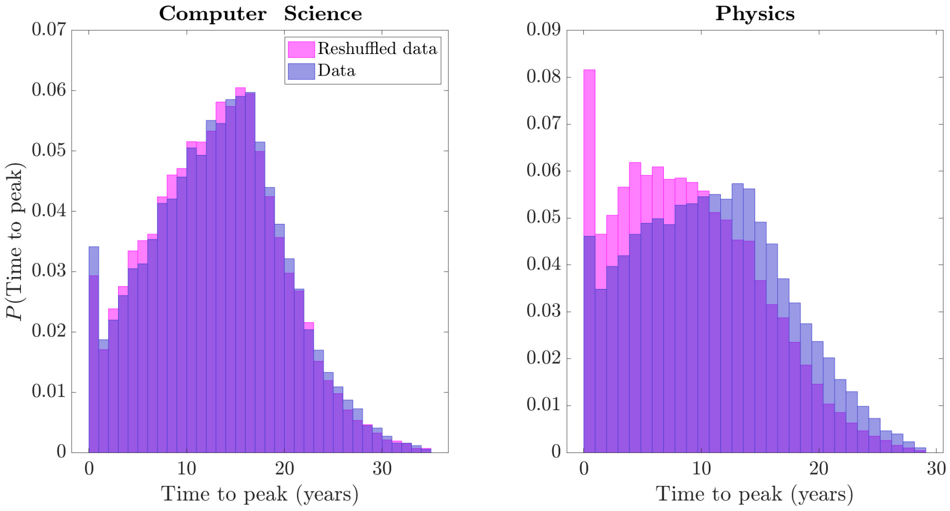

Supplementary Figure 3. Histograms of the number of years spent by researchers to reach their peak year in Computer Science (left) and Physics (right) based on the CD_5 metric (purple) and the randomized (magents) benchmark.

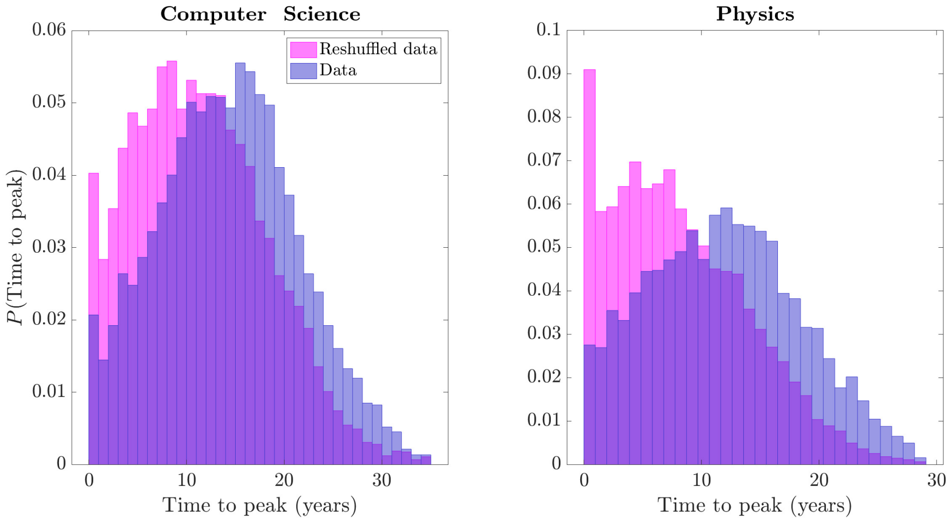

Supplementary Figure 4. Histograms of the number of years spent by researchers to reach their peak year in Computer Science (left) and Physics (right). The results are constructed with our pool of most disruptive researchers.

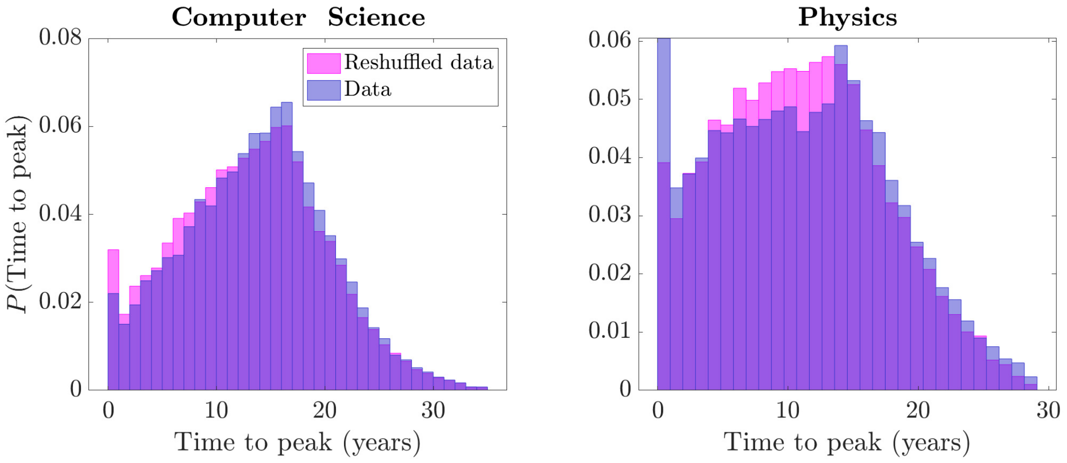

Supplementary Figure 5. Histograms of the number of years spent by researchers to reach their peak year in Computer Science (left) and Physics (right) based on the standardized disruption score (purple) and the randomized (pink) benchmark.

Supplementary Note 2 Characterizing peak year disruption: additional details

Here we present a series of figures and tables that provide further details on the comparison of disruption levels across the 'before peak year' (BPNY), 'peak year' (PY), and 'after peak year' (APNY) phases. We first report the statistical information for N_{CS} and N_{PHY} in the three career phases, as shown in Supplementary Table 1.

In the main text, we mention the size of the effect in the differences in disruption levels between the B/APNY and the PY periods. Supplementary Table 2 reports the precise values of our Cohen's d analysis. We also present the distribution plots of the number of papers published during the peak year and its comparison baseline in Supplementary Figure 6.

Furthermore, we validate our results in the main text with the following robustness checks. Firstly, we conduct the same comparison using the CD_5 metric and the standardized disruption score. The results of comparisons and their statistical counts and tests are illustrated in Supplementary Figure 7-8 and Supplementary Table 3-4, respectively. We then replicate the experiments with the pool of most disruptive researchers. Although the peak year disruption level of Computer Science (excluding the peak paper) is not the highest compared to the BPNY phases, our results remain consistent when we include the peak paper, as shown in Supplementary Figure 9. The statistical information for these comparisons are presented in Supplementary Table 5. Lastly, we collect papers in our dataset that contribute to the three career phases and directly compare the score distributions of these papers. As can be seen in Supplementary Figure 10 and Supplementary Table 6, we still observe an equivalent result.

	Computer Science					Physics				
	N_{CS}	KS (incl.)	KS (excl.)	MWU (incl.)	MWU (excl.)	N_{PHY}	KS (incl.)	KS (excl.)	MWU (incl.)	MWU (excl.)
BP5Y	23,495	0.000	0.000	0.000	0.000	31,138	0.000	0.000	0.000	0.000
BP4Y	23,037	0.000	0.000	0.000	0.000	31,056	0.000	0.000	0.000	0.000
BP3Y	22,308	0.000	0.000	0.000	0.002	30,258	0.000	0.000	0.000	0.000
BP2Y	20,980	0.000	0.000	0.000	0.043	28,719	0.000	0.000	0.000	0.000
PY (incl. peak paper)	27,641	-	-	-	-	34,526	-	-	-	-
PY (excl. peak paper)	18,640	-	-	-	-	26,164	-	-	-	-
AP2Y	21,987	0.000	0.000	0.000	0.000	30,441	0.000	0.000	0.000	0.000
AP3Y	23,473	0.000	0.000	0.000	0.000	32,166	0.000	0.000	0.000	0.000
AP4Y	24,338	0.000	0.000	0.000	0.000	33,094	0.000	0.000	0.000	0.000
AP5Y	24,876	0.000	0.000	0.000	0.000	33,196	0.000	0.000	0.000	0.000

Supplementary Table 1. Statistical counts and pairwise KS and MWU tests between BPNY/APNY and PY (both include and exclude the peak disruption paper) in Computer Science and Physics.

	Computer Science		Physics	
	PY (incl. peak paper)	PY (excl. peak paper)	PY (incl. peak paper)	PY (excl. peak paper)
BP5Y	0.658	0.011	0.535	0.030
BP4Y	0.654	0.008	0.534	0.029
BP3Y	0.649	0.007	0.531	0.030
BP2Y	0.640	0.004	0.525	0.031
AP2Y	0.659	0.034	0.536	0.043
AP3Y	0.670	0.036	0.543	0.045
AP4Y	0.678	0.041	0.549	0.050
AP5Y	0.683	0.042	0.551	0.056

Supplementary Table 2. Cohen's d computed with the disruption levels of PK and B/APNY for researchers in Computer Science and Physics. Here the disruption levels of PK are calculated excluding (left) and including (right) the peak paper.

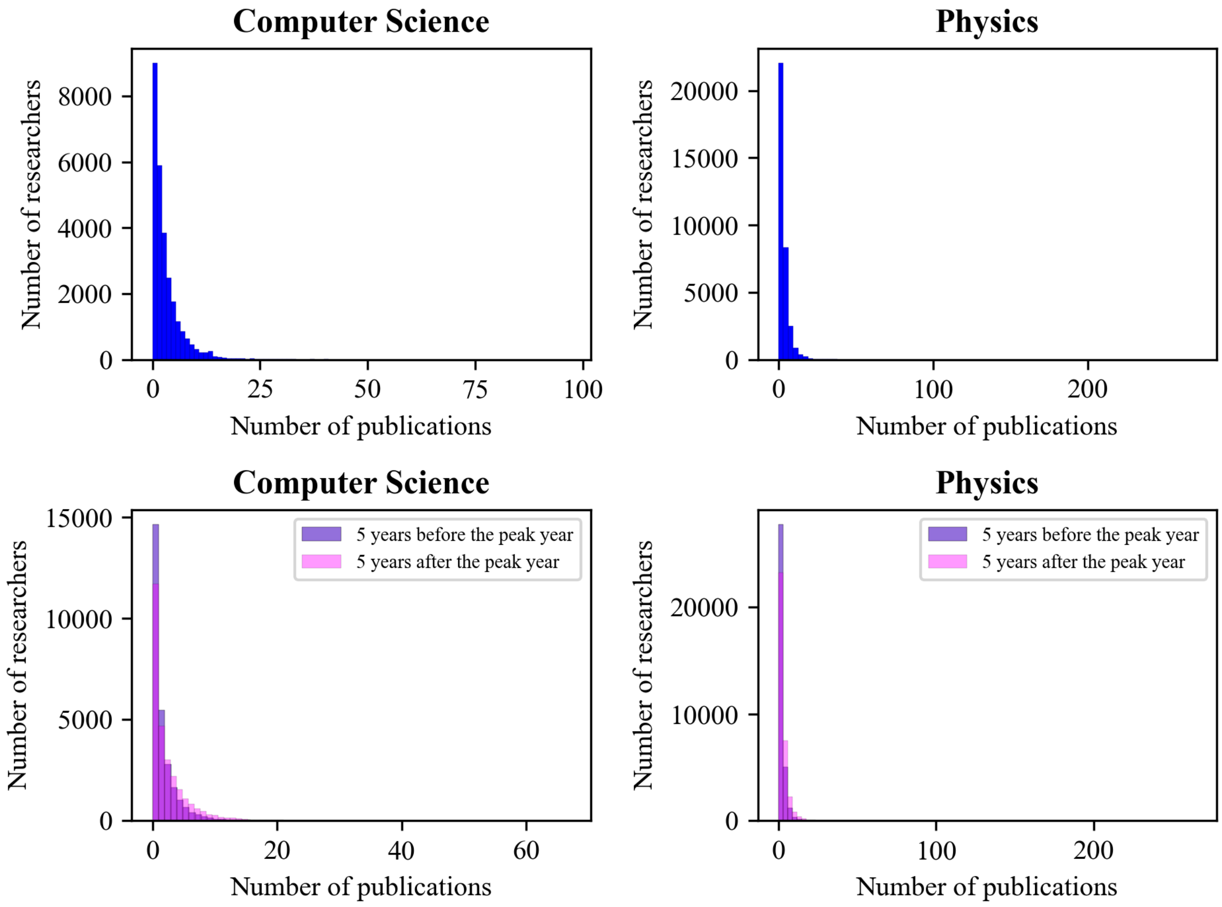

Supplementary Figure 6. Top row: Distribution of the number of papers published by researchers in Computer Science (left) and Physics (right) during the peak year. Bottom row: Distribution of the number of papers published five years before and after the peak year, which serve as a comparison baseline. It can be seen that both the peak year and comparison baseline distributions follow a power law distribution.

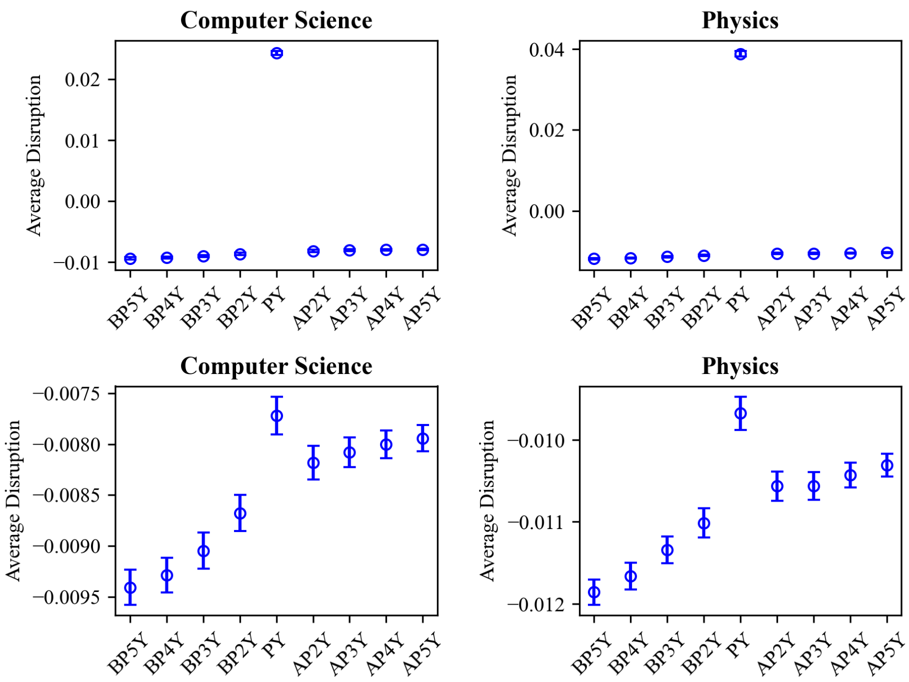

Supplementary Figure 7. Robustness check on the average disruption levels of the three career phases using the CD_5 metric. The PY phase is computed both with the peak disruption paper included (top row) and excluded (bottom row) from the analysis.

	Computer Science					Physics				
	N_{CS}	KS (incl.)	KS (excl.)	MWU (incl.)	MWU (excl.)	N_{PHY}	KS (incl.)	KS (excl.)	MWU (incl.)	MWU (excl.)
BP5Y	23,777	0.000	0.000	0.000	0.000	31,441	0.000	0.000	0.000	0.000
BP4Y	23,254	0.000	0.000	0.000	0.000	31,354	0.000	0.000	0.000	0.000
BP3Y	22,516	0.000	0.000	0.000	0.000	30,554	0.000	0.000	0.000	0.000
BP2Y	21,149	0.000	0.000	0.000	0.000	29,052	0.000	0.000	0.000	0.000
PY (incl. peak paper)	27,641	-	-	-	-	34,526	-	-	-	-
PY (excl. peak paper)	18,466	-	-	-	-	26,428	-	-	-	-
AP2Y	21,056	0.000	0.000	0.000	0.000	30,378	0.000	0.000	0.000	0.000
AP3Y	22,506	0.000	0.000	0.000	0.000	32,017	0.000	0.000	0.000	0.000
AP4Y	23,299	0.000	0.000	0.000	0.000	32,873	0.000	0.000	0.000	0.000
AP5Y	23,788	0.000	0.000	0.000	0.000	32,965	0.000	0.000	0.000	0.000

Supplementary Table 3. Robustness check with CD_5 metric. The table depicts statistical counts and pairwise KS and MWU tests between B/APNY and PY in both disciplines.

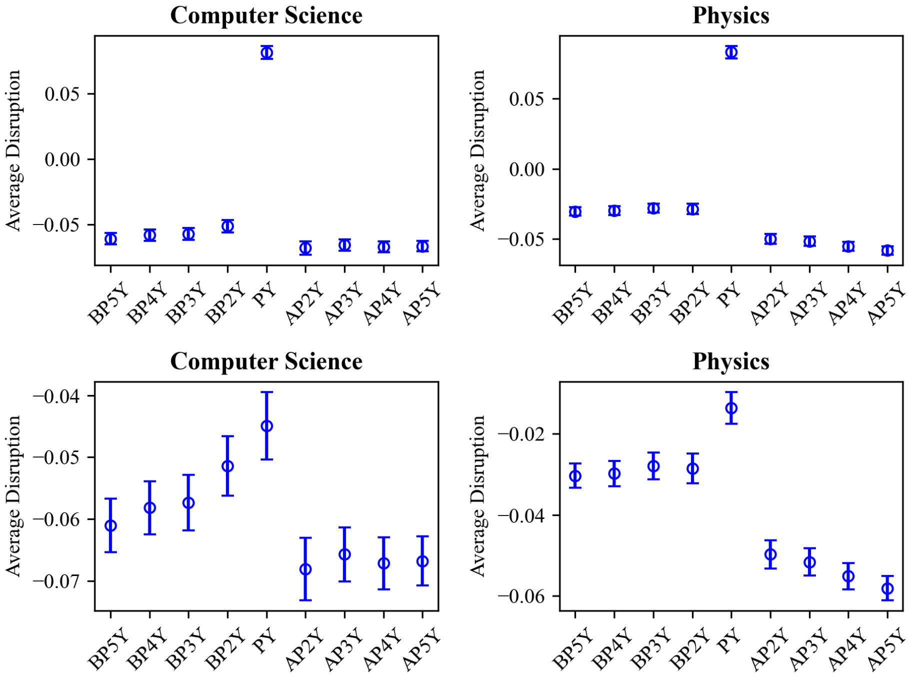

Supplementary Figure 8. Robustness check on the average disruption levels of the three career phases using the standardized disruption score. The PY phase is computed both with the peak disruption paper included (top row) and excluded (bottom row) from the analysis.

	Computer Science					Physics				
	N_{CS}	KS (incl.)	KS (excl.)	MWU (incl.)	MWU (excl.)	N_{PHY}	KS (incl.)	KS (excl.)	MWU (incl.)	MWU (excl.)
BP5Y	24,561	0.000	0.000	0.000	0.000	32,398	0.000	0.000	0.000	0.000
BP4Y	24,081	0.000	0.000	0.000	0.000	32,303	0.000	0.000	0.000	0.000
BP3Y	23,365	0.000	0.000	0.000	0.000	31,455	0.000	0.000	0.000	0.000
BP2Y	22,070	0.000	0.000	0.000	0.000	29,808	0.000	0.000	0.000	0.000
PY (incl. peak paper)	27,641	-	-	-	-	34,526	-	-	-	-
PY (excl. peak paper)	19,453	-	-	-	-	26,235	-	-	-	-
AP2Y	21,571	0.000	0.000	0.000	0.000	27,623	0.000	0.000	0.000	0.000
AP3Y	22,722	0.000	0.000	0.000	0.000	28,951	0.000	0.000	0.000	0.000
AP4Y	23,385	0.000	0.000	0.000	0.000	29,659	0.000	0.000	0.000	0.000
AP5Y	23,777	0.000	0.000	0.000	0.000	29,729	0.000	0.000	0.000	0.000

Supplementary Table 4. Robustness check with the standardized score. The table depicts statistical counts and pairwise KS and MWU tests between B/APNY and PY in both disciplines.

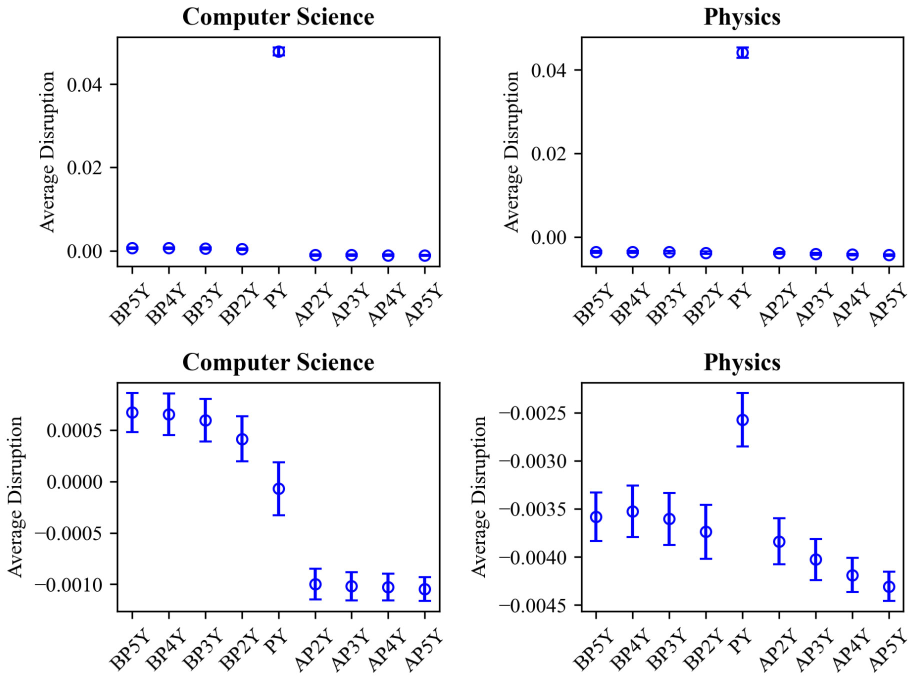

Supplementary Figure 9. Robustness check with the pool of most disruptive researchers. The PY phase is computed both with the peak disruption paper included (top row) and excluded (bottom row) from the analysis. While the PY in Computer Science (exclude the peak paper) is not the highest compared to the BPNY periods, our results still hold in Physics.

	Computer Science					Physics				
	N_{CS}	KS (incl.)	KS (excl.)	MWU (incl.)	MWU (excl.)	N_{PHY}	KS (incl.)	KS (excl.)	MWU (incl.)	MWU (excl.)
BP5Y	6,648	0.000	0.000	0.000	0.080	7,768	0.000	0.000	0.000	0.000
BP4Y	6,575	0.000	0.002	0.000	0.280	7,760	0.000	0.000	0.000	0.000
BP3Y	6,435	0.000	0.017	0.000	0.488	7,661	0.000	0.000	0.000	0.000
BP2Y	6,168	0.000	0.063	0.000	0.965	7,427	0.000	0.000	0.000	0.000
PY (incl. peak paper)	7,547	-	-	-	-	8,576	-	-	-	-
PY (excl. peak paper)	5,749	-	-	-	-	7,148	-	-	-	-
AP2Y	6,674	0.000	0.000	0.000	0.000	8,003	0.000	0.000	0.000	0.000
AP3Y	6,969	0.000	0.000	0.000	0.000	8,257	0.000	0.000	0.000	0.000
AP4Y	7,124	0.000	0.000	0.000	0.000	8,386	0.000	0.000	0.000	0.000
AP5Y	7,213	0.000	0.000	0.000	0.000	8,394	0.000	0.000	0.000	0.000

Supplementary Table 5. Statistical counts and pairwise KS and MWU tests for the pool of most disruptive researchers in both Computer Science and Physics.

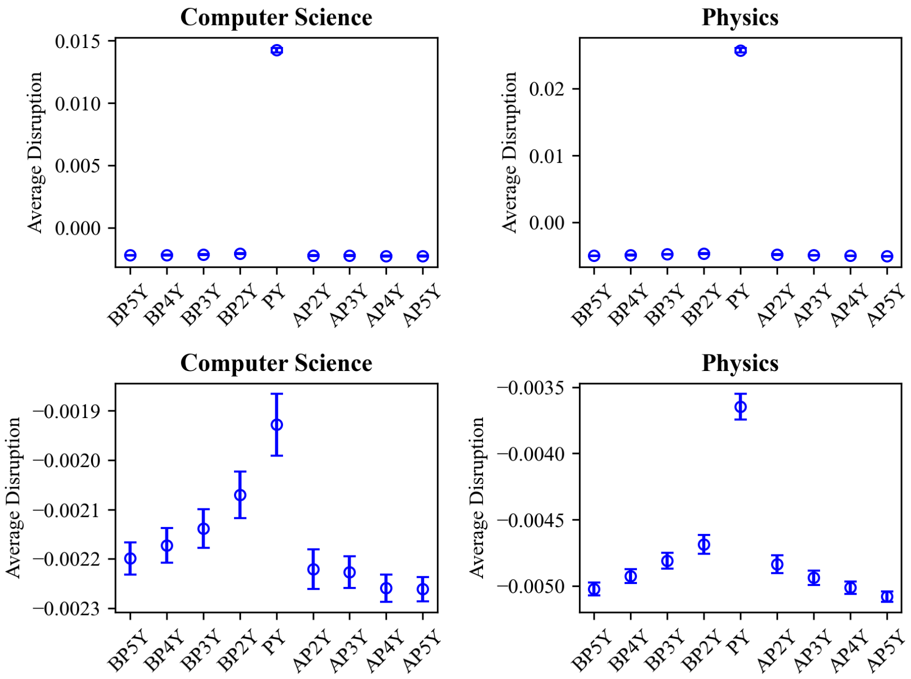

Supplementary Figure 10. Robustness check with distributions of papers. We collect papers contributing to the three career phases and compare their distributions. The PY phase is computed both with the peak disruption paper included (top row) and excluded (bottom row) from the analysis.

	Computer Science					Physics				
	N_{CS}	KS (incl.)	KS (excl.)	MWU (incl.)	MWU (excl.)	N_{PHY}	KS (incl.)	KS (excl.)	MWU (incl.)	MWU (excl.)
BP5Y	240,873	0.000	0.000	0.000	0.000	324,420	0.000	0.000	0.000	0.000
BP4Y	204,806	0.000	0.000	0.000	0.000	274,209	0.000	0.000	0.000	0.000
BP3Y	163,150	0.000	0.000	0.000	0.000	216,147	0.000	0.000	0.000	0.000
BP2Y	115,921	0.000	0.000	0.000	0.000	151,993	0.000	0.000	0.000	0.000
PK (incl. peak paper)	96,101	-	-	-	-	93,582	-	-	-	-
PK (excl. peak paper)	68,460	-	-	-	-	128,108	-	-	-	-
AP2Y	137,643	0.000	0.000	0.000	0.000	182,564	0.000	0.000	0.000	0.000
AP3Y	204,133	0.000	0.000	0.000	0.000	270,872	0.000	0.000	0.000	0.000
AP4Y	268,964	0.000	0.000	0.000	0.000	359,069	0.000	0.000	0.000	0.000
AP5Y	330,939	0.000	0.000	0.000	0.000	443,648	0.000	0.000	0.000	0.000

Supplementary Table 6. Robustness check with distributions of papers. The table depicts statistical counts and pairwise KS and MWU tests between B/APNY and PY in both disciplines.

Supplementary Note 3 Distribution plots of effort-related variables

To quantify the determinants of peak year disruption, we develop a series of linear models to measure the relationship between a researcher's disruption during their peak year and the relative effort, relative productivity, and relative time devotion associated to that year. Here, we show the distributions of such quantities in both Computer Science and Physics, which are plotted in Supplementary Figure 11 as normalized histograms. As it can be seen, the distributions for the (logarithm of) relative effort and relative productivity are very similar across disciplines, whereas the distributions for relative time devoted are much more irregular and display some differences.

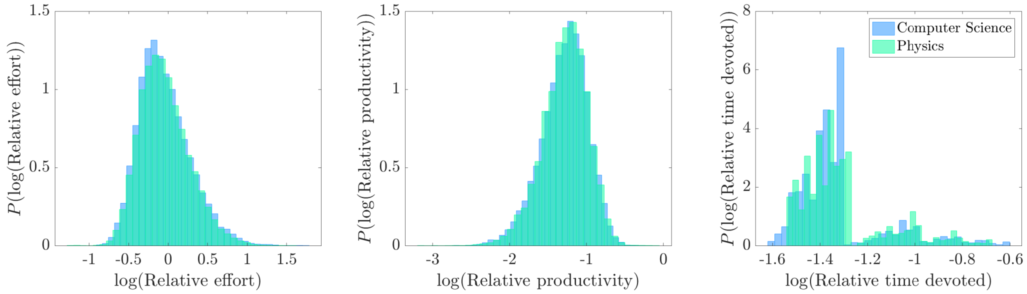

Supplementary Figure 11. Normalized histograms for the distributions of relative effort (left), relative productivity (center), and relative time devoted (right) associated to the peak year for researchers in our pool.

Supplementary Note 4 Additional regressions on scientific disruption and impact

In the main text, the regression results on the disruption and impact of peak year papers are obtained from variables associated with the peak year data (see Supplementary Tables 7-10). As a robustness check, we build linear models on the same dependent variable with regressors computed in both the peak year and the two years before the peak year phases, see Supplementary Tables 11-14. We also calibrate linear models with the dependent variable computed from CD_5, standardized disruption scores, and our pool of disruptive researchers, see Supplementary Tables 15-18, Supplementary Tables 19-22, and Supplementary Tables 23-26, respectively. Moreover, we include the logarithm of the average number of coauthors and the average number of references, i.e., log(coauthors) and log(references) as additional independent variables. The results are illustrated in Supplementary Tables 27-30.

In both disciplines, relative effort and relative time devotion contribute positively to the peak year disruption level, whereas relative productivity negatively affects the peak year disruption. In impact regressions, however, the same set of variables yield the opposite effect (the only exception is relative productivity in Computer Science when we construct the impact regression with additional log(coauthors) and log(references) variables), which captures the difference in the mechanics of scientific disruption and impact. Similar results can be found when considering the corresponding absolute quantities.

Supplementary Table 7. Regression results for peak year disruption levels in Computer Science obtained with the peak year data.

Indep. Variables	Computer Science: Models for peak year disruption levels					
	Model 1	Model 2	Model 3	Model 4	Model 5	Model 6
Relative effort	0.172***					
	(0.007)					
Effort		0.137***				
		(0.007)				
Relative productivity			-0.135***			
			(0.007)			
Productivity				-0.121***		
				(0.007)		
Relative time devoted					0.023***	
					(0.007)	
Time devoted						0.021***
						(0.007)
Avg. num. of coauthors	0.108***	0.099***	0.107***	0.103***	0.117***	0.117***
	(0.006)	(0.007)	(0.007)	(0.007)	(0.007)	(0.007)
Avg. prev. disruption	0.201***	0.209***	0.205***	0.209***	0.206***	0.206***
	(0.006)	(0.006)	(0.006)	(0.006)	(0.007)	(0.007)
Peak year	-0.251***	-0.256***	-0.221***	-0.256***	-0.285***	-0.272***
	(0.009)	(0.009)	(0.009)	(0.009)	(0.010)	(0.009)
Time to peak	0.087***	0.067***	0.040***	0.070***	0.070***	0.057***
	(0.009)	(0.009)	(0.009)	(0.009)	(0.010)	(0.009)
N	20,980	20,980	20,980	20,980	20,980	20,980
R^2	0.142	0.133	0.132	0.129	0.116	0.116

Supplementary Table 8. Regression results for peak year impact in Computer Science obtained with the peak year data.

Indep. Variables	Computer Science: Models for peak year impact					
	Model 1	Model 2	Model 3	Model 4	Model 5	Model 6
Relative effort	-0.056***					
	(0.007)					
Effort		-0.182***				
		(0.007)				
Relative productivity			0.021***			
			(0.007)			
Productivity				0.370***		
				(0.007)		
Relative time devoted					-0.077***	
					(0.008)	
Time devoted						-0.056***
						(0.007)
Avg. num. of coauthors	0.030***	0.051***	0.028***	0.072***	0.029***	0.028***
	(0.007)	(0.007)	(0.007)	(0.007)	(0.007)	(0.007)
Avg. prev. disruption	0.035***	0.029***	0.034***	0.023***	0.033***	0.034***
	(0.007)	(0.007)	(0.007)	(0.006)	(0.007)	(0.007)
Peak year	0.008	-0.007	0.008	-0.037***	0.058***	0.014
	(0.010)	(0.010)	(0.010)	(0.009)	(0.011)	(0.010)
Time to peak	0.044***	0.039***	0.056***	0.012	0.007	0.052***
	(0.010)	(0.009)	(0.010)	(0.009)	(0.011)	(0.010)
N	20,980	20,980	20,980	20,980	20,980	20,980
R^2	0.009	0.038	0.007	0.134	0.011	0.009

Supplementary Table 9. Regression results for peak year disruption in Physics obtained with the peak year data.

Indep. Variables	Physics: Models for peak year disruption levels					
	Model 1	Model 2	Model 3	Model 4	Model 5	Model 6
Relative effort	0.141***					
	(0.006)					
Effort		0.205***				
		(0.006)				
Relative productivity			-0.106***			
			(0.006)			
Productivity				-0.097***		
				(0.006)		
Relative time devoted					0.070***	
					(0.006)	
Time devoted						0.059***
						(0.006)
Avg. num. of coauthors	-0.004	0.007	-0.005	0.005	0.009	0.008
	(0.006)	(0.006)	(0.006)	(0.006)	(0.006)	(0.006)
Avg. prev. disruption	0.232***	0.229***	0.233***	0.233***	0.233***	0.233***
	(0.006)	(0.006)	(0.006)	(0.006)	(0.006)	(0.006)
Peak year	-0.143***	-0.152***	-0.118***	-0.149***	-0.187***	-0.150***
	(0.008)	(0.008)	(0.008)	(0.008)	(0.009)	(0.008)
Time to peak	0.095***	0.098***	0.059***	0.089***	0.114***	0.080***
	(0.008)	(0.008)	(0.008)	(0.008)	(0.009)	(0.008)
N	28,867	28,867	28,867	28,867	28,867	28,867
R^2	0.086	0.108	0.078	0.076	0.071	0.070

Supplementary Table 10. Regression results for peak year impact in Physics obtained with the peak year data.

Indep. Variables	Physics: Models for peak year impact					
	Model 1	Model 2	Model 3	Model 4	Model 5	Model 6
Relative effort	-0.018***					
	(0.006)					
Effort		-0.074***				
		(0.006)				
Relative productivity			0.010			
			(0.006)			
Productivity				0.157***		
				(0.006)		
Relative time devoted					-0.038***	
					(0.006)	
Time devoted						-0.029***
						(0.006)
Avg. num. of coauthors	0.006	0.005	0.006	0.005	0.003	0.004
	(0.006)	(0.006)	(0.006)	(0.006)	(0.006)	(0.006)
Avg. prev. disruption	-0.004	-0.003	-0.004	-0.004	-0.004	-0.004
	(0.006)	(0.006)	(0.006)	(0.006)	(0.006)	(0.006)
Peak year	-0.037***	-0.035***	-0.039***	-0.036***	-0.016*	-0.036***
	(0.008)	(0.008)	(0.009)	(0.008)	(0.009)	(0.008)
Time to peak	0.015*	0.010	0.019***	-0.003	-0.003	0.016*
	(0.008)	(0.008)	(0.008)	(0.008)	(0.009)	(0.008)
N	28,867	28,867	28,867	28,867	28,867	28,867
R^2	0.001	0.006	0.001	0.025	0.002	0.002

Supplementary Table 11. Regression results for peak year disruption levels in Computer Science obtained with the peak year data and the two years preceding the peak year data.

Indep. Variables	Computer Science: Models for peak year disruption levels					
	Model 1	Model 2	Model 3	Model 4	Model 5	Model 6
**Peak year variables**						
Relative effort	0.175***<br>(0.007)					
Effort		0.150***<br>(0.007)				
Relative productivity			-0.140***<br>(0.007)			
Productivity				-0.199***<br>(0.011)		
Relative time devoted					0.026***<br>(0.008)	
Time devoted						0.027***<br>(0.007)
Avg. num. of coauthors	0.091***<br>(0.008)	0.086***<br>(0.008)	0.086***<br>(0.008)	0.085***<br>(0.008)	0.104***<br>(0.008)	0.104***<br>(0.008)
**2 years before peak variables**						
Relative effort	-0.010<br>(0.007)					
Effort		-0.033***<br>(0.007)				
Relative productivity			0.016**<br>(0.007)			
Productivity				0.099***<br>(0.011)		
Relative time devoted					0.016**<br>(0.007)	
Time devoted						0.021***<br>(0.007)
Avg. num. of coauthors	0.047***<br>(0.008)	0.040***<br>(0.008)	0.055***<br>(0.008)	0.045***<br>(0.008)	0.040***<br>(0.008)	0.040***<br>(0.008)
**Control variables**						
Avg. prev. disruption	0.189***<br>(0.007)	0.198***<br>(0.007)	0.192***<br>(0.007)	0.197***<br>(0.007)	0.193***<br>(0.007)	0.193***<br>(0.007)
Peak year	-0.254***<br>(0.009)	-0.261***<br>(0.009)	-0.230***<br>(0.010)	-0.261***<br>(0.009)	-0.296***<br>(0.011)	-0.273***<br>(0.009)
Time to peak	0.086***<br>(0.009)	0.065***<br>(0.009)	0.044***<br>(0.009)	0.065***<br>(0.009)	0.084***<br>(0.011)	0.062***<br>(0.009)
N	20,081	20,081	20,081	20,081	20,081	20,081
R^2	0.140	0.132	0.129	0.130	0.113	0.114

Supplementary Table 12. Regression results for peak year impact in Computer Science obtained with the peak year data and the two years preceding the peak year data.

Indep. Variables	Computer Science: Models for peak year impact					
	Model 1	Model 2	Model 3	Model 4	Model 5	Model 6
**Peak year variables**						
Relative effort	-0.057*** (0.008)					
Effort		-0.154*** (0.008)				
Relative productivity			0.026*** (0.008)			
Productivity				0.292*** (0.011)		
Relative time devoted					-0.092*** (0.008)	
Time devoted						-0.074*** (0.007)
Avg. num. of coauthors	0.030*** (0.008)	0.041*** (0.008)	0.030*** (0.008)	0.055*** (0.008)	0.027*** (0.008)	0.026*** (0.008)
**2 years before peak variables**						
Relative effort	-0.002 (0.008)					
Effort		-0.076*** (0.008)				
Relative productivity			-0.021*** (0.008)			
Productivity				0.098*** (0.011)		
Relative time devoted					-0.073*** (0.008)	
Time devoted						-0.062*** (0.007)
Avg. num. of coauthors	0.0001 (0.008)	0.023*** (0.008)	-0.003 (0.008)	0.031*** (0.008)	0.010 (0.008)	0.009 (0.008)
**Other variables**						
Avg. prev. disruption	0.035*** (0.007)	0.027*** (0.007)	0.034*** (0.007)	0.020*** (0.007)	0.033*** (0.007)	0.034*** (0.007)
Peak year	0.005 (0.010)	-0.013 (0.009)	0.011 (0.010)	-0.042*** (0.009)	0.095*** (0.011)	0.008 (0.009)
Time to peak	0.039*** (0.010)	0.032*** (0.009)	0.050*** (0.009)	0.007 (0.009)	-0.044*** (0.012)	0.043*** (0.009)
N	20,081	20,081	20,081	20,081	20,081	20,081
R^2	0.008	0.042	0.006	0.137	0.015	0.012

Supplementary Table 13. Regression results for peak year disruption levels in Physics obtained with the peak year data and the two years preceding the peak year data.

Indep. Variables	Physics: Models for peak year disruption levels					
	Model 1	Model 2	Model 3	Model 4	Model 5	Model 6
**Peak year variables**						
Relative effort	0.154*** (0.006)					
Effort		0.203*** (0.006)				
Relative productivity			-0.105*** (0.006)			
Productivity				-0.109*** (0.009)		
Relative time devoted					0.086*** (0.007)	
Time devoted						0.080*** (0.007)
Avg. num. of coauthors	-0.017** (0.007)	0.001 (0.007)	-0.017** (0.007)	-0.004 (0.007)	0.006 (0.007)	0.006 (0.007)
**2 years before peak var.**						
Relative effort	-0.027*** (0.007)					
Effort		0.007 (0.006)				
Relative productivity			0.004 (0.006)			
Productivity				0.018 (0.010)		
Relative time devoted					0.062*** (0.007)	
Time devoted						0.056*** (0.007)
Avg. num. of coauthors	0.017** (0.007)	0.010 (0.007)	0.019** (0.007)	0.012 (0.007)	0.003 (0.007)	0.003 (0.007)
**Other variables**						
Avg. prev. disruption	0.248*** (0.006)	0.245*** (0.006)	0.250*** (0.006)	0.250*** (0.006)	0.247*** (0.006)	0.248*** (0.006)
Peak year	-0.137*** (0.008)	-0.146*** (0.008)	-0.114*** (0.009)	-0.141*** (0.008)	-0.214*** (0.010)	-0.140*** (0.008)
Time to peak	0.091*** (0.008)	0.094*** (0.008)	0.062*** (0.008)	0.088*** (0.008)	0.151*** (0.010)	0.084*** (0.008)
N	25,222	25,222	25,222	25,222	25,222	25,222
R^2	0.095	0.116	0.084	0.083	0.081	0.080

Supplementary Table 14. Regression results for peak year impact in Physics obtained with the peak year data and the two years preceding the peak year data.

Indep. Variables	Physics: Models for peak year impact					
	Model 1	Model 2	Model 3	Model 4	Model 5	Model 6
**Peak year variables**						
Relative effort	-0.034*** (0.007)					
Effort		-0.080*** (0.007)				
Relative productivity			0.021*** (0.007)			
Productivity				0.124*** (0.010)		
Relative time devoted					-0.057*** (0.007)	
Time devoted						-0.046*** (0.007)
Avg. num. of coauthors	0.014 (0.007)	0.004 (0.007)	0.014 (0.007)	-0.001 (0.007)	0.005 (0.007)	0.006 (0.007)
**2 years before peak variables**						
Relative effort	0.010 (0.007)					
Effort		-0.038*** (0.007)				
Relative productivity			-0.013 (0.007)			
Productivity				0.123*** (0.010)		
Relative time devoted					-0.034*** (0.007)	
time devoted						-0.026*** (0.007)
Avg. num. of coauthors	0.0004 (0.007)	0.006 (0.007)	-0.002 (0.008)	0.011 (0.007)	0.006 (0.007)	0.006 (0.007)
**Other variables**						
Avg. prev. disruption	-0.007 (0.006)	-0.004 (0.006)	-0.007 (0.006)	-0.006 (0.006)	-0.006 (0.006)	-0.007 (0.006)
Peak year	-0.038*** (0.009)	-0.035*** (0.009)	-0.039*** (0.009)	-0.038*** (0.008)	0.008 (0.010)	-0.037*** (0.009)
Time to peak	0.014 (0.009)	0.007 (0.008)	0.019** (0.009)	-0.013 (0.008)	-0.028*** (0.010)	0.014 (0.008)
N	25,222	25,222	25,222	25,222	25,222	25,222
R^2	0.002	0.011	0.001	0.054	0.004	0.003

Supplementary Table 15. Regression results for peak year disruption levels in Computer Science, measured with the CD_5 metric.

Indep. Variables	Computer Science: Models for peak year disruption levels (CD_5)					
	Model 1	Model 2	Model 3	Model 4	Model 5	Model 6
Relative effort	0.188***					
	(0.007)					
Effort		0.191***				
		(0.007)				
Relative productivity			-0.160***			
			(0.007)			
Productivity				-0.171***		
				(0.007)		
Relative time devotion					0.034***	
					(0.008)	
Time devotion						0.030***
						(0.007)
Avg. num. of coauthors	0.161***	0.157***	0.161***	0.159***	0.161***	0.162***
	(0.007)	(0.007)	(0.007)	(0.007)	(0.007)	(0.007)
Avg. prev. disruption	0.042***	0.051***	0.043***	0.052***	0.046***	0.046***
	(0.007)	(0.007)	(0.007)	(0.006)	(0.007)	(0.007)
Peak year	-0.149***	-0.150***	-0.116***	-0.150***	-0.193***	-0.172***
	(0.009)	(0.009)	(0.010)	(0.009)	(0.010)	(0.010)
Time to peak	0.093***	0.078***	0.036***	0.080***	0.086***	0.065***
	(0.009)	(0.009)	(0.009)	(0.009)	(0.011)	(0.009)
N	21,149	21,149	21,149	21,149	21,149	21,149
R^2	0.075	0.077	0.065	0.069	0.043	0.043

Supplementary Table 16. Regression results for peak year impact in Computer Science, for the CD_5 robustness check.

Indep. Variables	Computer Science: Models for peak year impact					
	Model 1	Model 2	Model 3	Model 4	Model 5	Model 6
Relative effort	-0.052***					
	(0.007)					
Effort		-0.151***				
		(0.007)				
Relative productivity			0.023***			
			(0.007)			
Productivity				0.327***		
				(0.007)		
Relative time devotion					-0.067***	
					(0.008)	
Time devotion						-0.047***
						(0.007)
Avg. num. of coauthors	0.049***	0.053***	0.049***	0.054***	0.049***	0.049***
	(0.007)	(0.007)	(0.007)	(0.007)	(0.007)	(0.007)
Avg. prev. disruption	0.022***	0.017**	0.022***	0.009	0.021***	0.021***
	(0.007)	(0.007)	(0.007)	(0.007)	(0.007)	(0.007)
Peak year	0.003	-0.009	0.001	-0.036***	0.046***	0.007
	(0.010)	(0.010)	(0.010)	(0.009)	(0.011)	(0.010)
Time to peak	0.046***	0.043***	0.058***	0.023**	0.010	0.053***
	(0.010)	(0.010)	(0.010)	(0.009)	(0.011)	(0.010)
N	21,149	21,149	21,149	21,149	21,149	21,149
R^2	0.010	0.029	0.008	0.109	0.011	0.010

Supplementary Table 17. Regression results for peak year disruption levels in Physics, measured with the CD_5 metric.

Indep. Variables	Physics: Models for peak year disruption levels (CD_5)					
	Model 1	Model 2	Model 3	Model 4	Model 5	Model 6
Relative effort	0.172***					
	(0.006)					
Effort		0.221***				
		(0.006)				
Relative productivity			-0.116***			
			(0.006)			
Productivity				-0.103***		
				(0.006)		
Relative time devotion					0.075***	
					(0.006)	
Time devotion						0.064***
						(0.006)
Avg. num. of coauthors	-0.022***	0.009	-0.026***	-0.016***	-0.025***	-0.025***
	(0.006)	(0.006)	(0.006)	(0.006)	(0.006)	(0.006)
Avg. prev. disruption	0.160***	0.161***	0.163***	0.162***	0.162***	0.162***
	(0.006)	(0.006)	(0.006)	(0.006)	(0.006)	(0.006)
Peak year	-0.160***	-0.175***	-0.130***	-0.165***	-0.204***	-0.164***
	(0.008)	(0.008)	(0.008)	(0.008)	(0.009)	(0.009)
Time to peak	0.094***	0.098***	0.048***	0.084***	0.112***	0.073***
	(0.008)	(0.008)	(0.008)	(0.008)	(0.009)	(0.008)
N	29,052	29,052	29,052	29,052	29,052	29,052
R^2	0.069	0.087	0.053	0.050	0.045	0.044

Supplementary Table 18. Regression results for peak year impact in Physics, for the CD_5 robustness check.

Indep. Variables	Physics: Models for peak year impact					
	Model 1	Model 2	Model 3	Model 4	Model 5	Model 6
Relative effort	-0.083***					
	(0.006)					
Effort		-0.210***				
		(0.006)				
Relative productivity			0.064***			
			(0.006)			
Productivity				0.695***		
				(0.004)		
Relative time devotion					-0.091***	
					(0.006)	
Time devotion						-0.073***
						(0.006)
Avg. num. of coauthors	0.084***	0.051***	0.085***	-0.006	0.081***	0.082***
	(0.006)	(0.006)	(0.006)	(0.004)	(0.006)	(0.006)
Avg. prev. disruption	0.002	0.001	0.002	-0.006	0.001	0.000
	(0.006)	(0.006)	(0.006)	(0.004)	(0.006)	(0.006)
Peak year	0.038***	0.051***	0.021**	0.058***	0.090***	0.041***
	(0.008)	(0.008)	(0.009)	(0.006)	(0.009)	(0.008)
Time to peak	0.066***	0.051***	0.089***	-0.061***	0.026***	0.074***
	(0.008)	(0.008)	(0.008)	(0.006)	(0.009)	(0.008)
N	29,052	29,052	29,052	29,052	29,052	29,052
R^2	0.027	0.063	0.025	0.488	0.028	0.026

Supplementary Table 19. Regression results for peak year disruption levels in Computer Science, measured with the standardized disruption score.

Indep. Variables	Computer Science: Models for peak year disruption levels (std. score)					
	Model 1	Model 2	Model 3	Model 4	Model 5	Model 6
Relative effort	0.179***					
	(0.007)					
Effort		0.142***				
		(0.006)				
Relative productivity			-0.143***			
			(0.007)			
Productivity				-0.127***		
				(0.006)		
Relative time devoted					0.025***	
					(0.007)	
Time devoted						0.025***
						(0.006)
Avg. num. of coauthors	0.184***	0.180***	0.188***	0.185***	0.198***	0.198***
	(0.006)	(0.006)	(0.006)	(0.006)	(0.006)	(0.006)
Avg. prev. innovation	0.148***	0.155***	0.150***	0.155***	0.151***	0.151***
	(0.006)	(0.006)	(0.006)	(0.006)	(0.006)	(0.006)
Peak year	-0.180***	-0.190***	-0.151***	-0.189***	-0.218***	-0.203***
	(0.009)	(0.009)	(0.009)	(0.009)	(0.010)	(0.009)
Time to peak	0.056***	0.036***	0.005	0.040***	0.046***	0.031***
	(0.009)	(0.009)	(0.009)	(0.009)	(0.010)	(0.009)
N	22,070	22,070	22,070	22,070	22,070	22,070
R^2	0.126	0.116	0.116	0.112	0.097	0.097

Supplementary Table 20. Regression results for peak year impact in Computer Science, for the robustness check of standardized disruption scores.

Indep. Variables	Computer Science: Models for peak year impact					
	Model 1	Model 2	Model 3	Model 4	Model 5	Model 6
Relative effort	-0.065***					
	(0.007)					
Effort		-0.182***				
		(0.007)				
Relative productivity			0.030***			
			(0.007)			
Productivity				0.381***		
				(0.006)		
Relative time devoted					-0.083***	
					(0.008)	
Time devoted						-0.060***
						(0.007)
Avg. num. of coauthors	0.031***	0.051***	0.029***	0.069***	0.029***	0.028***
	(0.007)	(0.007)	(0.007)	(0.006)	(0.007)	(0.007)
Avg. prev. disruption	0.029***	0.022***	0.028***	0.014**	0.027***	0.028***
	(0.007)	(0.007)	(0.007)	(0.006)	(0.007)	(0.007)
Peak year	-0.015	-0.024***	-0.017**	-0.051***	0.039***	-0.008
	(0.009)	(0.009)	(0.010)	(0.009)	(0.010)	(0.009)
Time to peak	0.028***	0.031***	0.042***	0.009	-0.015***	0.036***
	(0.009)	(0.009)	(0.009)	(0.009)	(0.010)	(0.009)
N	22,070	22,070	22,070	22,070	22,070	22,070
R^2	0.007	0.035	0.004	0.142	0.008	0.006

Supplementary Table 21. Regression results for peak year disruption levels in Physics, measured with the standardized disruption score.

Indep. Variables	Physics: Models for peak year disruption levels (std. score)					
	Model 1	Model 2	Model 3	Model 4	Model 5	Model 6
Relative effort	0.128***					
	(0.006)					
Effort		0.183***				
		(0.006)				
Relative productivity			-0.096***			
			(0.006)			
Productivity				-0.084***		
				(0.006)		
Relative time devoted					0.064***	
					(0.006)	
Time devoted						0.057***
						(0.006)
Avg. num. of coauthors	-0.012**	-0.002	-0.012**	-0.004	-0.002	-0.002
	(0.006)	(0.006)	(0.006)	(0.006)	(0.006)	(0.006)
Avg. prev. disruption	0.216***	0.213***	0.217***	0.217***	0.217***	0.218***
	(0.006)	(0.005)	(0.006)	(0.006)	(0.006)	(0.006)
Peak year	-0.128***	-0.141***	-0.103***	-0.135***	-0.170***	-0.133***
	(0.009)	(0.009)	(0.009)	(0.009)	(0.010)	(0.009)
Time to peak	0.035***	0.032***	-0.003	0.031***	0.064***	0.027***
	(0.009)	(0.009)	(0.009)	(0.009)	(0.010)	(0.009)
N	29,978	29,978	29,978	29,978	29,978	29,978
R^2	0.077	0.094	0.070	0.068	0.064	0.064

Supplementary Table 22. Regression results for peak year impact in Physics, for the robustness check of standardized disruption scores.

Indep. Variables	Physics: Models for peak year impact					
	Model 1	Model 2	Model 3	Model 4	Model 5	Model 6
Relative effort	-0.097***					
	(0.006)					
Effort		-0.226***				
		(0.006)				
Relative productivity			0.081***			
			(0.006)			
Productivity				0.705***		
				(0.004)		
Relative time devoted					-0.105***	
					(0.006)	
Time devoted						-0.085***
						(0.006)
Avg. num. of coauthors	0.041***	0.032***	0.042***	0.032***	0.031***	0.032***
	(0.006)	(0.006)	(0.006)	(0.004)	(0.006)	(0.006)
Avg. prev. disruption	-0.015***	-0.011*	-0.016***	-0.012***	-0.016***	-0.016***
	(0.006)	(0.006)	(0.006)	(0.004)	(0.006)	(0.006)
Peak year	0.025***	0.039***	0.004	0.052***	0.091***	0.029***
	(0.009)	(0.009)	(0.009)	(0.006)	(0.010)	(0.009)
Time to peak	0.019**	0.018**	0.050***	-0.024***	-0.038***	0.023***
	(0.009)	(0.009)	(0.009)	(0.006)	(0.010)	(0.009)
N	29,978	29,978	29,978	29,978	29,978	29,978
R^2	0.014	0.055	0.011	0.501	0.014	0.011

Supplementary Table 23. Regression results for peak year disruption levels in Computer Science obtained with the pool of disruptive researchers.

Indep. Variables	Computer Science: Models for peak year disruption levels					
	Model 1	Model 2	Model 3	Model 4	Model 5	Model 6
Relative effort	0.181***					
	(0.012)					
Effort		0.260***				
		(0.012)				
Relative productivity			-0.158***			
			(0.012)			
Productivity				-0.196***		
				(0.012)		
Relative time devotion					0.114***	
					(0.014)	
Time devotion						0.084***
						(0.012)
Avg. num. of coauthors	0.171***	0.149***	0.169***	0.159***	0.169***	0.169***
	(0.012)	(0.012)	(0.012)	(0.012)	(0.012)	(0.012)
Avg. prev. disruption	0.262***	0.257***	0.269***	0.265***	0.267***	0.267***
	(0.011)	(0.011)	(0.012)	(0.012)	(0.012)	(0.012)
Peak year	-0.243***	-0.212***	-0.199***	-0.214***	-0.334***	-0.254***
	(0.016)	(0.016)	(0.017)	(0.016)	(0.019)	(0.016)
Time to peak	0.100***	0.091***	0.048***	0.085***	0.148***	0.066***
	(0.016)	(0.016)	(0.016)	(0.016)	(0.019)	(0.016)
N	6,183	6,183	6,183	6,183	6,183	6,183
R^2	0.189	0.221	0.181	0.193	0.168	0.166

Supplementary Table 24. Regression results for peak year impact in Computer Science obtained with the pool of disruptive researchers.

Indep. Variables	Computer Science: Models for peak year impact					
	Model 1	Model 2	Model 3	Model 4	Model 5	Model 6
Relative effort	-0.059***					
	(0.013)					
Effort		-0.131***				
		(0.013)				
Relative productivity			0.063***			
			(0.013)			
Productivity				0.291***		
				(0.013)		
Relative time devotion					-0.044***	
					(0.015)	
Time devotion						-0.026**
						(0.013)
Avg. num. of coauthors	0.031**	0.042***	0.032**	0.049***	0.032**	0.031**
	(0.013)	(0.013)	(0.013)	(0.012)	(0.013)	(0.013)
Avg. prev. disruption	0.008	0.012	0.006	0.010	0.007	0.007
	(0.013)	(0.013)	(0.013)	(0.012)	(0.013)	(0.013)
Peak year	0.143***	0.124***	0.124***	0.082***	0.177***	0.147***
	(0.018)	(0.018)	(0.018)	(0.017)	(0.020)	(0.018)
Time to peak	-0.013	-0.015	0.006	-0.034**	-0.033	-0.002
	(0.018)	(0.018)	(0.018)	(0.017)	(0.021)	(0.018)
N	6,183	6,183	6,183	6,183	6,183	6,183
R^2	0.027	0.040	0.028	0.100	0.025	0.025

Supplementary Table 25. Regression results for peak year disruption levels in Physics obtained with the pool of disruptive researchers.

Indep. Variables	Physics: Models for peak year disruption levels					
	Model 1	Model 2	Model 3	Model 4	Model 5	Model 6
Relative effort	0.170***					
	(0.011)					
Effort		0.287***				
		(0.011)				
Relative productivity			-0.113***			
			(0.011)			
Productivity				-0.089***		
				(0.011)		
Relative time devotion					0.112***	
					(0.013)	
Time devotion						0.092***
						(0.011)
Avg. num. of coauthors	-0.054***	-0.013	-0.057***	-0.054***	-0.056***	-0.057***
	(0.011)	(0.011)	(0.011)	(0.011)	(0.011)	(0.011)
Avg. prev. disruption	0.200***	0.196***	0.204***	0.204***	0.201***	0.203***
	(0.011)	(0.011)	(0.011)	(0.011)	(0.011)	(0.011)
Peak year	-0.180***	-0.180***	-0.145***	-0.171***	-0.245***	-0.177***
	(0.016)	(0.015)	(0.016)	(0.016)	(0.018)	(0.016)
Time to peak	0.084***	0.098***	0.034**	0.061***	0.119***	0.055***
	(0.016)	(0.015)	(0.016)	(0.016)	(0.018)	(0.016)
N	7,427	7,427	7,427	7,427	7,427	7,427
R^2	0.101	0.150	0.085	0.080	0.082	0.081

Supplementary Table 26. Regression results for peak year impact in Physics obtained with the pool of disruptive researchers.

Indep. Variables	Physics: Models for peak year impact					
	Model 1	Model 2	Model 3	Model 4	Model 5	Model 6
Relative effort	-0.071***					
	(0.012)					
Effort		-0.186***				
		(0.012)				
Relative productivity			0.069***			
			(0.012)			
Productivity				0.756***		
				(0.008)		
Relative time devotion					-0.082***	
					(0.013)	
Time devotion						-0.063***
						(0.011)
Avg. num. of coauthors	0.067***	0.038***	0.067***	-0.009	0.066***	0.066***
	(0.012)	(0.012)	(0.012)	(0.008)	(0.012)	(0.012)
Avg. prev. disruption	-0.004	-0.001	-0.006	-0.014*	-0.004	-0.005
	(0.011)	(0.011)	(0.011)	(0.008)	(0.011)	(0.011)
Peak year	0.129***	0.130***	0.108***	0.085***	0.177***	0.127***
	(0.016)	(0.016)	(0.016)	(0.017)	(0.018)	(0.016)
Time to peak	0.053***	0.035**	0.076***	-0.050***	0.016	0.063***
	(0.016)	(0.016)	(0.016)	(0.011)	(0.018)	(0.016)
N	7,427	7,427	7,427	7,427	7,427	7,427
R^2	0.047	0.075	0.047	0.582	0.048	0.046

Supplementary Table 27. Regression results for peak year disruption levels in Computer Science with log(coauthors) and log(references) as additional independent variables.

Indep. Variables	Computer Science: Models for peak year disruption levels					
	Model 1	Model 2	Model 3	Model 4	Model 5	Model 6
Relative effort	0.167***					
	(0.007)					
Effort		0.124***				
		(0.007)				
Relative productivity			-0.146***			
			(0.007)			
Productivity				-0.106***		
				(0.007)		
Relative time devotion					0.017**	
					(0.007)	
Time devotion						0.016**
						(0.007)
log(avg. num. of coauthors)	0.003	0.010	-0.001	0.002	-0.006	-0.006
	(0.007)	(0.007)	(0.007)	(0.007)	(0.007)	(0.007)
log(avg. num. of references)	-0.240***	-0.228***	-0.250***	-0.233***	-0.256***	-0.256***
	(0.007)	(0.007)	(0.007)	(0.007)	(0.007)	(0.007)
Avg. prev. disruption	0.130***	0.136***	0.131***	0.137***	0.134***	0.134***
	(0.006)	(0.006)	(0.006)	(0.006)	(0.007)	(0.007)
Peak year	-0.174***	-0.183***	-0.137***	-0.181***	-0.199***	-0.189***
	(0.009)	(0.009)	(0.010)	(0.009)	(0.010)	(0.009)
Time to peak	0.106***	0.088***	0.057***	0.089***	0.090***	0.079***
	(0.009)	(0.009)	(0.009)	(0.010)	(0.010)	(0.009)
N	20,718	20,718	20,718	20,718	20,718	20,718
R^2	0.150	0.138	0.144	0.134	0.125	0.125

Supplementary Table 28. Regression results for peak year impact in Computer Science with log(coauthors) and log(references) as additional independent variables.

Indep. Variables	Computer Science: Models for peak year impact					
	Model 1	Model 2	Model 3	Model 4	Model 5	Model 6
Relative effort	-0.015**					
	(0.007)					
Effort		-0.104***				
		(0.007)				
Relative productivity			-0.005			
			(0.007)			
Productivity				0.302***		
				(0.007)		
Relative time devotion					-0.049***	
					(0.008)	
Time devotion						-0.031***
						(0.007)
log(avg. num. of coauthors)	0.051***	0.038***	0.052***	0.027***	0.050***	0.050***
	(0.007)	(0.007)	(0.007)	(0.007)	(0.007)	(0.007)
log(avg. num. of references)	0.241***	0.218***	0.243***	0.174***	0.239***	0.241***
	(0.007)	(0.007)	(0.007)	(0.007)	(0.007)	(0.007)
Avg. prev. disruption	0.040***	0.037***	0.039***	0.030***	0.039***	0.039***
	(0.007)	(0.007)	(0.007)	(0.006)	(0.007)	(0.007)
Peak year	-0.044***	-0.048***	-0.041***	-0.067***	-0.014	-0.044***
	(0.010)	(0.010)	(0.010)	(0.009)	(0.011)	(0.010)
Time to peak	0.034***	0.029***	0.036***	0.007	0.005	0.036***
	(0.010)	(0.009)	(0.010)	(0.009)	(0.011)	(0.010)
N	20,718	20,718	20,718	20,718	20,718	20,718
R^2	0.065	0.074	0.065	0.146	0.066	0.065

Supplementary Table 29. Regression results for peak year disruption levels in Physics with log(coauthors) and log(references) as additional independent variables.

Indep. Variables	Physics: Models for peak year disruption levels					
	Model 1	Model 2	Model 3	Model 4	Model 5	Model 6
Relative effort	0.105***					
	(0.005)					
Effort		0.143***				
		(0.006)				
Relative productivity			-0.084***			
			(0.005)			
Productivity				-0.062***		
				(0.005)		
Relative time devotion					0.035***	
					(0.006)	
Time devotion						0.032***
						(0.005)
log(avg. num. of coauthors)	-0.075***	-0.043***	-0.080***	-0.077***	-0.085***	-0.085***
	(0.005)	(0.006)	(0.005)	(0.005)	(0.005)	(0.005)
log(avg. num. of references)	-0.346***	-0.336***	-0.356***	-0.357***	-0.359***	-0.360***
	(0.006)	(0.006)	(0.006)	(0.006)	(0.006)	(0.006)
Avg. prev. disruption	0.198***	0.199***	0.198***	0.198***	0.198***	0.198***
	(0.005)	(0.005)	(0.005)	(0.005)	(0.005)	(0.005)
Peak year	-0.053***	-0.065***	-0.027***	-0.051***	-0.068***	-0.049***
	(0.008)	(0.008)	(0.008)	(0.008)	(0.008)	(0.008)
Time to peak	0.100***	0.102***	0.070***	0.093***	0.105***	0.087***
	(0.008)	(0.007)	(0.008)	(0.008)	(0.008)	(0.008)
N	28,719	28,719	28,719	28,719	28,719	28,719
R^2	0.210	0.218	0.207	0.204	0.201	0.201

Supplementary Table 30. Regression results for peak year impact in Physics with log(coauthors) and log(references) as additional independent variables.

Indep. Variables	Physics: Models for peak year impact					
	Model 1	Model 2	Model 3	Model 4	Model 5	Model 6
Relative effort	-0.038***					
	(0.006)					
Effort		-0.159***				
		(0.006)				
Relative productivity			0.034***			
			(0.006)			
Productivity				0.699***		
				(0.004)		
Relative time devotion					-0.069***	
					(0.006)	
Time devotion						-0.054***
						(0.006)
log(avg. num. of coauthors)	0.117***	0.071***	0.118***	-0.008**	0.114***	0.115***
	(0.006)	(0.006)	(0.006)	(0.004)	(0.006)	(0.006)
log(avg. num. of references)	0.152***	0.129***	0.155***	0.106***	0.154***	0.155***
	(0.006)	(0.006)	(0.006)	(0.003)	(0.006)	(0.006)
Avg. prev. disruption	0.011*	0.010*	0.011*	0.011*	0.011*	0.011*
	(0.006)	(0.006)	(0.006)	(0.004)	(0.006)	(0.006)
Peak year	-0.019**	-0.001	-0.029***	0.014**	0.019**	-0.018**
	(0.008)	(0.008)	(0.009)	(0.006)	(0.009)	(0.008)
Time to peak	0.050***	0.037***	0.062***	-0.032***	0.018**	0.053***
	(0.008)	(0.007)	(0.008)	(0.006)	(0.009)	(0.008)
N	28,719	28,719	28,719	28,719	28,719	28,719
R^2	0.048	0.069	0.048	0.509	0.051	0.050
